# GRASP65 controls the cis Golgi integrity in vivo

**DOI:** 10.1242/bio.20147757

**Published:** 2014-05-02

**Authors:** Tineke Veenendaal, Tim Jarvela, Adam G. Grieve, Johan H. van Es, Adam D. Linstedt, Catherine Rabouille

**Affiliations:** 1 Hubrecht Institute-KNAW and University Medical Center Utrecht, Uppsalalaan 8, 3584 CT Utrecht, The Netherlands; 2 Department of Biological Sciences, Carnegie Mellon University, Pittsburgh, PA 15213, USA; 3 Department of Cell Biology, University Medical Center Utrecht, Heidelberglaan 100, 3584 CX Utrecht, The Netherlands; * Present address: Sir William Dunn School of Pathology, University of Oxford, Oxford OX1 3RE, UK.

**Keywords:** Knock-in mouse, GRASP65, GORASP, GRASP55, FRAP, Golgi, Glycosylation, Expression pattern, Golgi ribbon linking

## Abstract

GRASP65 and GRASP55 are peripheral Golgi proteins localized to cis and medial/trans cisternae, respectively. They are implicated in diverse aspects of protein transport and structure related to the Golgi complex, including the stacking of the Golgi stack and/or the linking of mammalian Golgi stacks into the Golgi ribbon. Using a mouse model, we interfered with GRASP65 by homologous recombination and confirmed its absence of expression. Surprisingly, the mice were healthy and fertile with no apparent defects in tissue, cellular or subcellular organization. Immortalized MEFs derived from the mice did not show any growth or morphological defects. However, despite the normal appearance of the Golgi ribbon, a fluorescence recovery after photobleaching assay revealed functional discontinuities specific to the cis cisternal membrane network. This leads to a strong change in the plasma membrane GSII lectin staining that was also observed in certain mutant tissues. These findings substantiate the role of GRASP65 in continuity of the cis Golgi network required for proper glycosylation, while showing that neither this continuity nor GRASP65 itself are essential for the viability of a complex organism.

## INTRODUCTION

GRASP65 (also called GORASP1) belongs to the GRASP family of myristoylated peripheral membrane proteins of the Golgi (Barr et al., 1997). GRASP65 has a very close homologue GRASP55 (GORASP2) that shares a high level of identity and similarity with GRASP65, especially in the N-terminus comprising the two PDZ domains (Truschel et al., 2011), whereas the C-terminus is not conserved (Shorter et al., 1999). GRASP65 is recruited to the cis cisternae of the Golgi stack through binding to GM130 and Rab1, whereas GRASP55 is recruited to the medial/trans Golgi cisternae through Golgin45 and Rab2 (Short et al., 2001).

The two mammalian members of this family, GRASP65 and GRASP55 were originally described as Golgi stacking factors in vitro (Barr et al., 1997; Shorter et al., 1999), but whether they have a similar role in vivo remains inconclusive. When GRASP65 was inactivated by antibody injection in NRK cells, this led to Golgi cisternae unstacking (Wang et al., 2003). Although knockdown of either GRASP65 (Sütterlin et al., 2002; Puthenveedu et al., 2006) or GRASP55 (Feinstein and Linstedt, 2008; Duran et al., 2008) by siRNA (or shRNA) in HeLa cells has been reported to not significantly unstack the Golgi cisternae, their combined depletion does (Xiang et al., 2013). In contrast, Golgi membranes remain stacked after depletion of the single GRASP orthologue in *Drosophila* (Kondylis et al., 2005) or in Trypanosomes (Vinke et al., 2011).

Mammalian GRASP65 and/or GRASP55 have also been shown to be important for Golgi ribbon integrity. Loss of function of either, whether by siRNA depletion (Puthenveedu et al., 2006; Feinstein and Linstedt, 2008) or inactivation by Killer-red (Jarvela and Linstedt, 2014) induces Golgi ribbon unlinking, which is fragmentation at the sites where small membrane tubules connect adjacent “mini-stacks” into a ribbon-like membrane network. Importantly, Killer-red inactivation of GRASP65 leads to unlinking of the cis cisternae, whereas inactivation of GRASP55 leads to unlinking of the trans side of the stack, in line with the Golgi sub-localization of the two GRASPs (Jarvela and Linstedt, 2014). This is particularly relevant as Golgi ribbon unlinking has been proposed to act as a checkpoint for cell entry into mitosis and is driven in part by phosphorylation of both GRASP65 and 55 during G2 phase (Duran et al., 2008; Feinstein and Linstedt, 2008; Persico et al., 2009; Rabouille and Kondylis, 2007; Sütterlin et al., 2002). GRASP-mediated control of Golgi linking appears to occur during Golgi repositioning upon directed cell migration and during Golgi fragmentation upon apoptosis (reviewed by Vinke et al., 2011).

Surprisingly, although GRASP family members do not seem to have a role in bulk transport of proteins through the Golgi, they have been recently linked to unconventional secretion of cytoplasmic and transmembrane proteins, two types of secretion that do not involve the Golgi (Nickel and Rabouille, 2009; Rabouille et al., 2012). dGRASP and GRASP65/55 have been shown to be involved in the Golgi bypass of Drosophila alpha-PS1 integrin and CFTR, respectively. The GRASP homologue in *Dictyostelium* and yeast, as well as GRASP55 in human macrophages, appears to be required for the unconventional secretion of cytoplasmic AcbA and IL1-beta, respectively (Rabouille et al., 2012). Interestingly, GRASP mediated unconventional secretion seems to be triggered by stress (either mechanical or nutritional) (Giuliani et al., 2011).

**Fig. 1.**
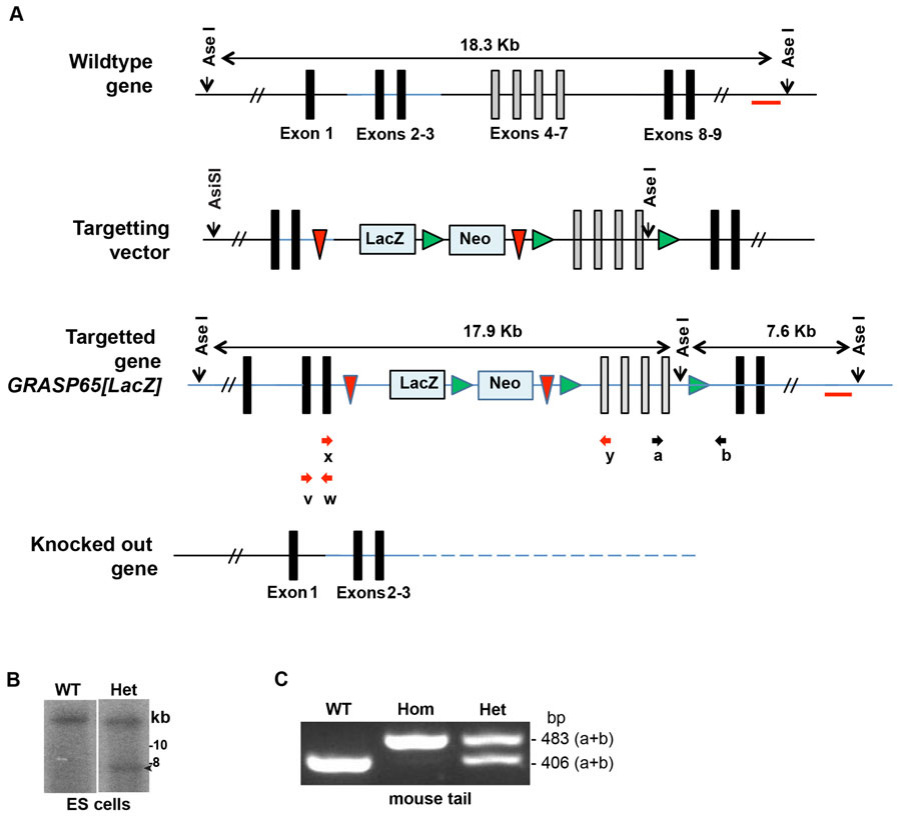
Generating *GRASP65* knock-in mouse. (A) Schematic representation of the wild type GRASP65/GORASP1 gene comprising exons 1–9. The targeting vector (from KOM) containing a LacZ, PGK Neo cassette flanked by FRT sites (red arrow heads) and the Lox (green arrow heads) flanking exons 4–7 (grey bars) (find full sequence in supplementary material Fig. S1); the final targeted gene in the chromosome of ES by homologous recombination; and the KO gene. The targeted GRASP65 locus harbours an extra AseI site resulting in a 7.6 kb fragment as opposed to the 18.3 kbp WT allele when detected with a 3′ flanking probe (indicated by a red line). The red bar below the WT GRASP65 gene represents the probe used for the Southern blot (see below). (B) Southern blot of AseI-digested genomic DNA from wild type (WT) and targeted heterozygous (Het) ES cells probed with a radioactive PCR fragment indicated in panel A (red bar, 656 bp) was designed to anneal outside of the 3′ homology arm. Note the single band at 18.3 kb in the wildtype cells and the additional 7.6 kb fragment (indicated by an arrow) in the heterozygous ES cells demonstrating site specific integration of the targeting construct. Mw (Kb) are indicated. (C) Genotyping PCR using genomic DNA from wild type/parental (WT), *GRASP65[LacZ]* homozygous (Hom) and heterozygous (Het) mouse tails using the primer combination “a” and “b” around the FRT site to distinguish between the wild type and targeted gene (see panel A and Table 1). These amplify a 406 bp fragment in the wildtype copy of the chromosome (WT and Het animals) and a 483 bp fragment in the targeted chromosome (Het and Hom animals).

To test which of these roles are fulfilled by GRASP65 in a mammalian animal, we generated a *GRASP65[LacZ]* knock-in mouse by homologous recombination that disrupts expression of the endogenous GRASP65 gene (see Materials and Methods; Fig. 1; supplementary material Fig. S1; Table 1). Unexpectedly, the block of *GRASP65* expression did not cause a noticeable phenotype at the organismal or tissue level. Even Golgi membranes evident in tissue sections and in isolated MEF cells were indistinguishable from the stacked and linked Golgi membranes of control samples. Nevertheless, a test of Golgi ribbon integrity using FRAP indicated significant unlinking of the cis but not trans Golgi cisternae and this was accompanied by glycosylation defects. Thus, GRASP65 mediates continuity of the cis Golgi network in vivo but it is not essential.

**Table 1.**
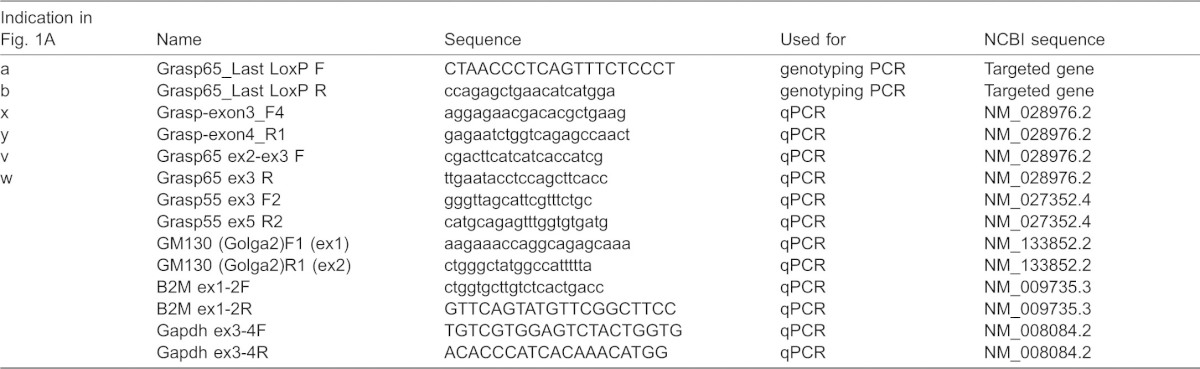
Primers used in this study

## RESULTS

### GRASP65 pattern of expression

To study the role of GRASP65 during development and adult homeostasis, we set out to make a knock-out mouse using Cre-LoxP strategy. To do so, we first targeted the GRASP65 locus by homologous recombination using a targeting vector containing LacZ-LoxP sites (*GRASP65[LacZ]*) (Fig. 1A) that was inserted to the *GRASP65* locus between exon 3 and 4.

This insertion leads to disruption in *GRASP65* full-length gene expression (Fig. 3A). Due to preferential splicing, only exon 1–3 and LacZ are expressed together under the control of the *GRASP65* promoter, but because of the IRES (internal ribosome entry site) sequences upstream of the LacZ start codon (Skarnes et al., 2011), exon 1–3 and LacZ are expressed independently of one another.

**Fig. 2.**
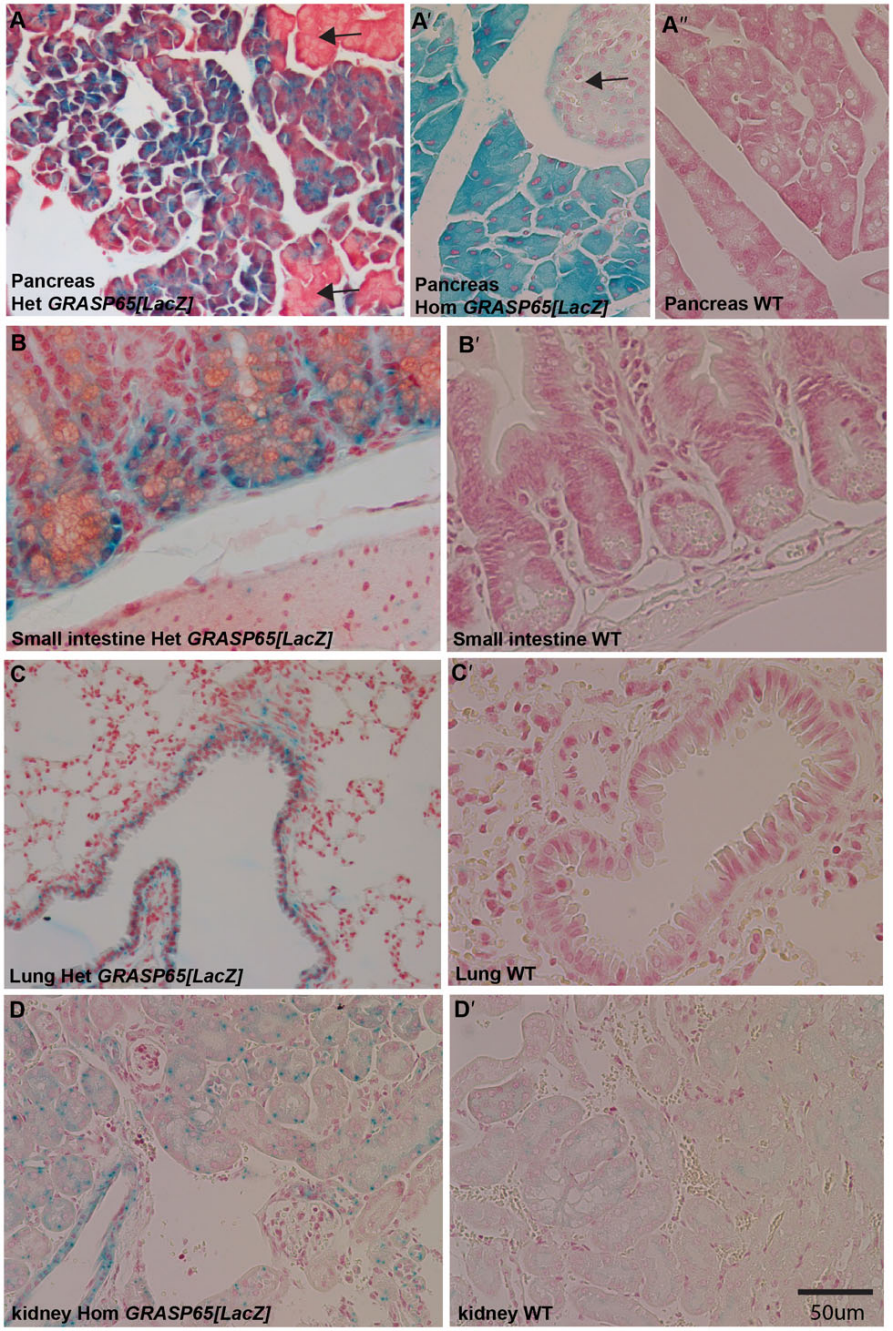
*GRASP65[LacZ]* expression in different tissues. (A–A″) LacZ staining of *GRASP65[LacZ]* heterozygous (Het, A), *GRASP65[LacZ]* homozygous (Hom, A′) and wildtype (WT, A″) pancreas. Note the total absence of staining in the wildtype and the specific LacZ staining in the exocrine pancreas. (B,B′) LacZ staining of heterozygous (Het, B), and wild type (WT, B′) small intestine. Note the specificity of staining to the crypt. (C,C′) LacZ staining of heterozygous (Het, C), and wild type (WT, C′) lungs. Note the specificity of staining to the clara cells. (D,D′) LacZ staining of heterozygous (Het, D), and wildtype (WT, D′) kidney. Note the specificity of staining as the WT does not display any staining at all. Scale bar: 50 µm.

**Fig. 3.**
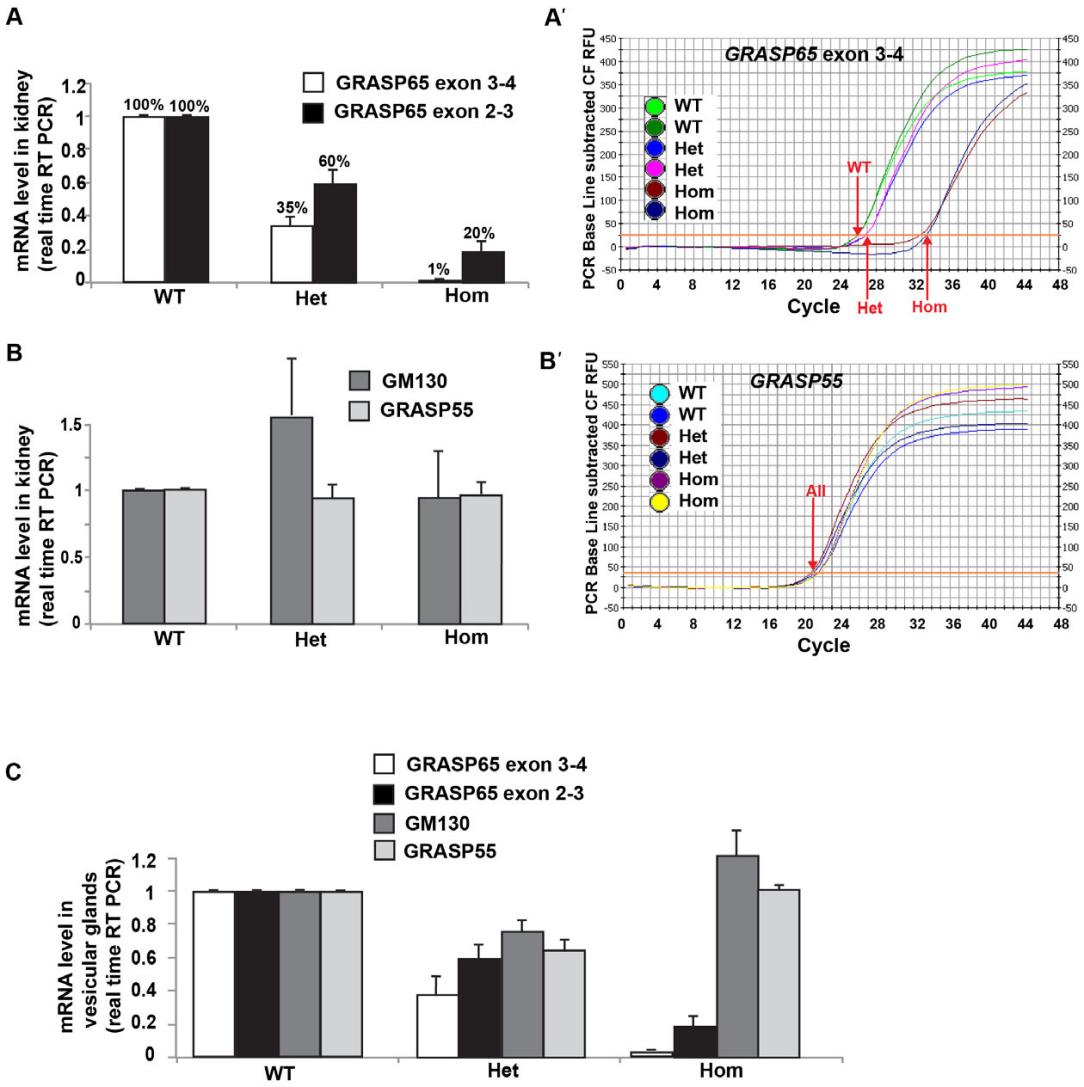
mRNA expression in *GRASP65[LacZ]* mice. (A,A′) *GRASP65* exon 2–3 and exon 3–4 mRNA expression monitored by real-time RT-qPCR using primers x and y (Fig. 1A) from wild-type (WT), *GRASP65[LacZ]* heterozygous (Het) and homozygous (Hom) mouse kidneys. This is derived from the number of cycles necessary to amplify above the background (red arrows in A′: 26 in WT, 27 in Het and 33.5 Hom). The lack of amplification in *GRASP65[LacZ]* Hom kidney indicates successful excision of all exons after the inserted LacZ. (B,B′) *GM130 and* GRASP55 mRNA expression monitored by real-time RT-qPCR using primers (Table 1) in the same kidneys as in panel A. Note that *GRASP55* level of expression is similar in the kidneys from mice of the three genotype (B′) as shown by the number of amplification cycles that do not change (21). (C) *GRASP65* exon 2–3 and exon 3–4, *GM130 and* GRASP55 mRNA expression monitored by real-time RT-qPCR using the same primers as in panels A and B.

Therefore, to examine GRASP65 expression pattern, we used heterozygous (and homozygous, see below) knock-in mice carrying one (or two) copies of the GRASP65 targeting vector expressing LacZ under the control of GRASP65 promoter (*GRASP65[LacZ]*) (Fig. 1A). Several tissues were fixed and processed for XGal staining before paraffin embedding, sectioning and analysis by light microscopy. GRASP65 is expressed in exocrine pancreas (but not islet of Langerhans) (Fig. 2A–A″), in the crypt of small (Fig. 2B,B′) and large (not shown) intestines, lung (especially in Clara cells, Fig. 2C,C′), kidney (Fig. 2D,D′), spleen and brain (not shown). Although the staining is low, it is specific, as it is totally absent in all wild type tissue sections (from the same litter) processed in parallel.

### GRASP65 loss of function does not lead to a phenotype in mice

To assess whether absence of *GRASP65* leads to an embryonic or adult phenotype, we crossed the *GRASP65[LacZ]* knock-in heterozygous mouse to homozygosity. The homozygous offspring is viable in a Mendelian ratio (Table 2), suggesting that GRASP65 does not have a major developmental role during embryogenesis. Adults also develop normally and their longevity is similar (∼57 weeks). We then tested their fertility by crossing a homozygous *GRASP65[LacZ]* female to a wild type male and a homozygous *GRASP65[LacZ]* male to a wild type female, both leading to generation of offspring in a Mendelian ratio (not shown). Finally, a homozygous *GRASP65[LacZ]* male crossed to a homozygous *GRASP65[LacZ]* female also led to offspring, demonstrating that loss of GRASP65 function has no effect on development, viability or fertility (Table 2). Because the *GRASP65[LacZ]* knock-in homozygous mice did not show any developmental or adult phenotype, we did not pursue with removing exons 4–7 by floxing the mice.

**Table 2.**
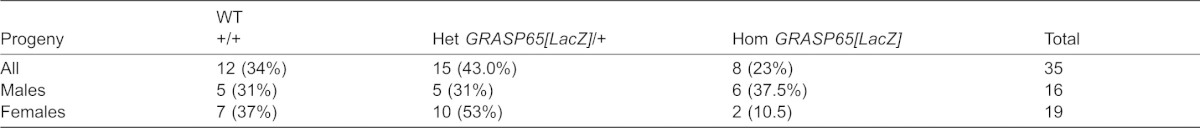
Mendelian ratio of progeny of *GRASP65[LacZ]*/+ × *GRASP65[LacZ]*/+

We confirmed GRASP65 loss of expression in *GRASP65[LacZ]* homozygous mice by real-time RT-qPCR. We showed that in kidney (Fig. 3A,A′) and vesicular glands (Fig. 3D) of these homozygous mice, exon 3–4 expression is reduced to 1% of the wild-type level.

As mentioned above, in theory, expression of exons 1–3 mRNA (encoding the first PDZ domain of GRASP65, supplementary material Fig. S2) (Truschel et al., 2011) should not be affected (Fig. 1A, targeted gene). However, when measured by real-time RT-qPCR, we find that exons 1–3 expression in kidney (Fig. 3B) and vesicular glands (Fig. 3E) of heterozygote and homozygous *GRASP65[LacZ]* mice is decreased to 60% and 20% of the wild-type level, respectively. This suggests that the exon 1–3 mRNA is either not efficiently transcribed or unstable.

**Fig. 4.**
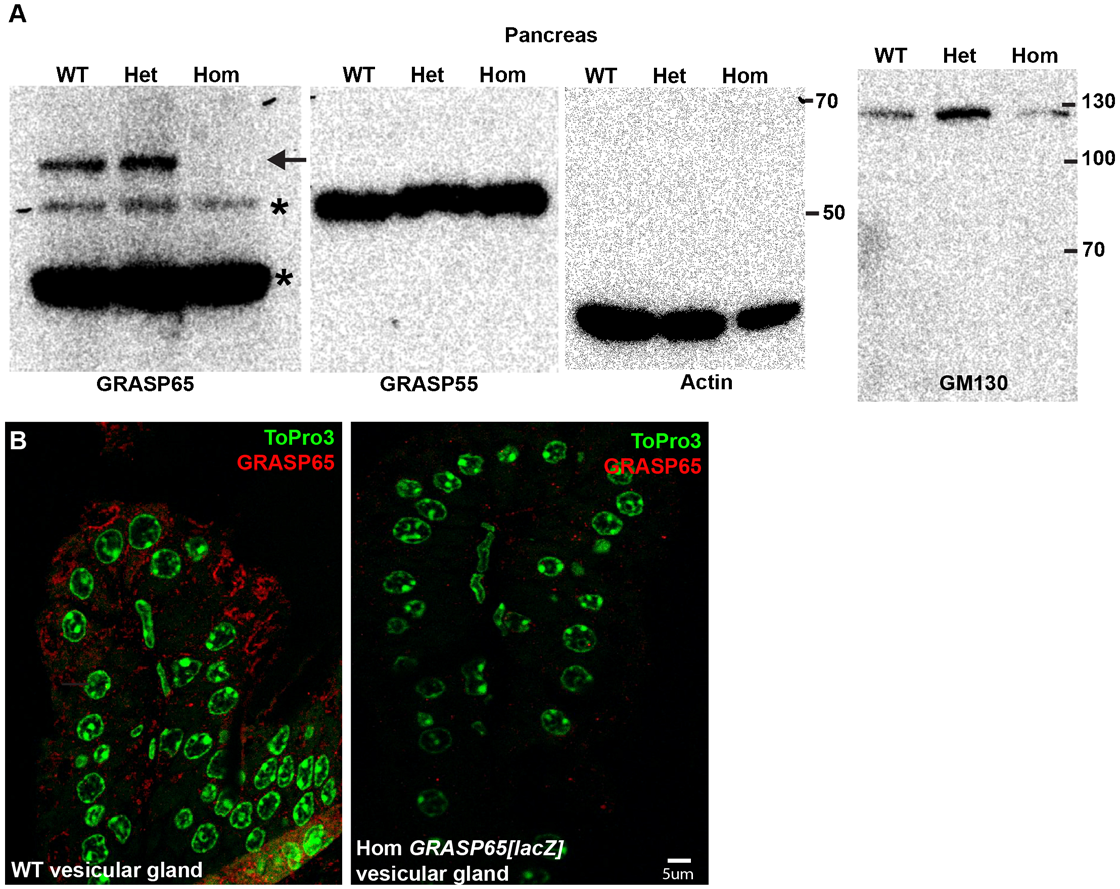
Protein expression in *GRASP65[LacZ]* mice. (A) Western blot of GRASP65, GRASP55, GM130 and actin in pancreas from wildtype (WT), *GRASP65[LacZ]* heterozygous (Het) and homozygous (Hom) mice using antibodies listed in Materials and Methods. Note that whereas GRASP65 is absent, GRASP55 is present at the same level in all tissues. GM130 expression is higher in heterozygous pancreas in agreement with the observed increase in mRNA expression in heterozygous kidney. The background bands are marked by an asterisk. (B) Immunofluorescence detection of GRASP65 (red, using FV18) in thick sections of wild type (WT) and *GRASP65[LacZ]* homozygous (Hom) vesicular glands. ToPro marks the nucleus. Note that GRASP65 staining is absent in the homozygous glands. Scale bar: 5 µm.

We then tested the loss of GRASP65 protein expression by Western blot and show that the protein is absent in homozygous pancreas (Fig. 4A) and vesicular glands (Fig. 4B). Overall, the homozygous *GRASP65[LacZ]* mouse is devoid of full length GRASP65 RNA and protein and expresses exon 1–3 mRNA to 20% of the wild type level that does not seem to be expressed as a detectable peptide.

### The Golgi apparatus of *GRASP65[LacZ]* homozygous mouse tissues does not appear to be structurally affected

**Fig. 5.**
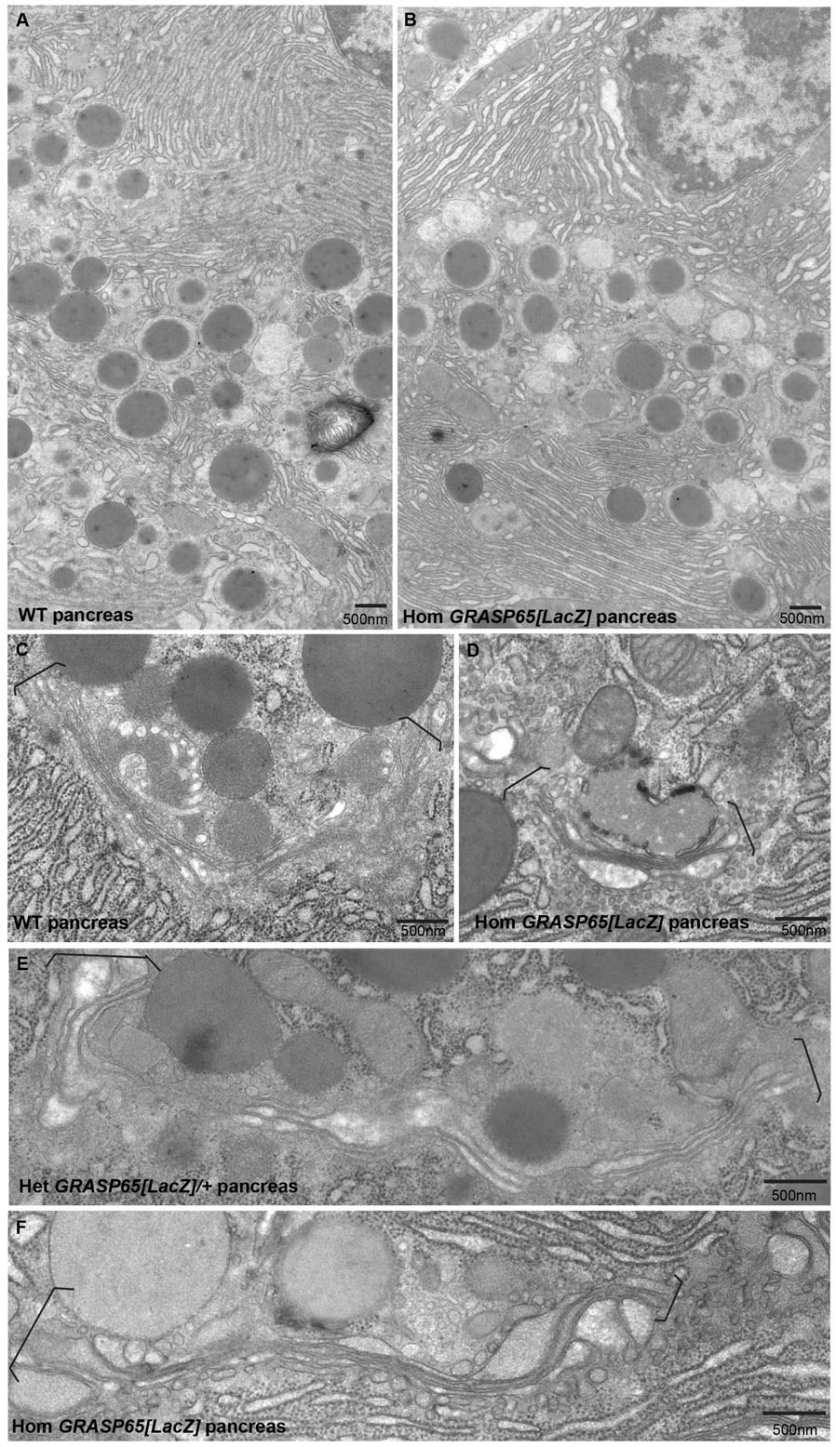
The Golgi apparatus appears normal in pancreas from *GRASP65[LacZ]* homozygous mice. (A,B) Low magnification view of exocrine pancreas in ultrathin epon sections from WT (A) and *GRASP65[LacZ]* homozygous (B) mice processed for conventional EM. (C–F) High magnification view of Golgi profiles (between brackets) in ultrathin epon sections of exocrine pancreas from WT (A), *GRASP65[LacZ]* het (C) and hom (D,F) mice. Note that no differences are detectable. Scale bars: 500 nm.

We further examined tissues (lung, small intestine, pancreas, vesicular glands etc.) dissected from wildtype, heterozygous and homozygous *GRASP65[LacZ]* mice for changes in their overall morphology, but could not detect any defects, in line with mouse viability. We further processed exocrine pancreas and vesicular glands for conventional electron microscopy to assess defects at (sub)-cellular level. Both tissues of the three genotypes appear similar at the ultrastructural level in terms of cell size, overall morphology, number, size and content of secretory granules, general morphology of the ER and cell–cell junction (Fig. 5A,A′ for pancreas and supplementary material Fig. S3A,B for vesicular glands).

With respect to the proposed role of GRASP65 in stacking Golgi cisternae and in Golgi ribbon integrity (Vinke et al., 2011), we examined Golgi morphology on EM sections. Both in wild type pancreas and vesicular glands, the Golgi ribbon is prominent, with large sections of the organelle as stacked cisternae linked into a ribbon (Fig. 5A,C for pancreas and supplementary material Fig. S2B for vesicular glands). This architecture is found very similar in heterozygous and homozygous *GRASP65[LacZ]* tissues (Fig. 5A,C; supplementary material Fig. S3B) and overall, we cannot detect specific and quantifiable defects in terms of cisternal stacking or ribbon organization (Fig. 5B,D; supplementary material Fig. S3C,D; Table 3).

**Table 3.**
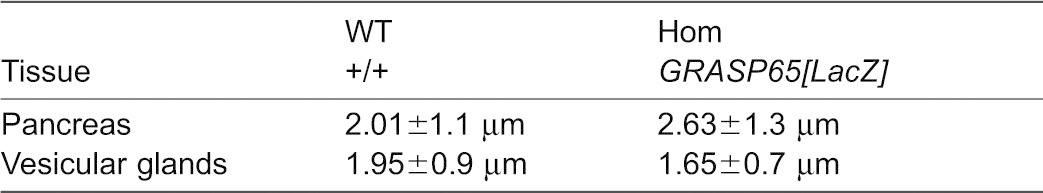
Measurement of Golgi ribbon length

### The loss of GRASP65 is not compensated by increased expression of GM130 and GRASP55

We then tested whether loss of GRASP65 is compensated by overexpression of GM130, the known GRASP65 receptor at the Golgi, and GRASP55, the second member of the GRASP family in mammals, which could explain the absence of Golgi defects. To do so, we measured GM130 and GRASP55 mRNAs by real-time RT-qPCR (Fig. 3) and proteins by Western Blot (Fig. 4) in the same kidneys (RT-qPCR) and pancreas (WB) that were tested for GRASP65. We did not find any significant difference in GRASP55 mRNA (Fig. 3D,D′) or protein (Fig. 4A) level in the *GRASP65[LacZ]* heterozygous and homozygous tissues when compared to wildtype from the same litter. We conclude that loss of GRASP65 does not result in overexpression of GRASP55.

This analysis reveals, however, that the *GRASP55* transcript is much more abundant in mouse tissues than *GRASP65* (compare the 21 cycles necessary for measuring *GRASP55* mRNA amplification (Fig. 3D′) to the 27 for *GRASP65* (Fig. 3A′), in complete agreement with the UNIGENE EST data on NCBI (http://www.ncbi.nlm.nih.gov/UniGene/clust.cgi?UGID = 546426&TAXID = 10090&SEARCH = GORASP1). However, whereas UNIGENE reported a lack of GRASP55 EST in vesicular glands, our data show that GRASP55 is actually expressed in this tissue (Fig. 3C).

For GM130, both the mRNA (Fig. 3B, kidney) and protein level (Fig. 4A, pancreas) are slightly higher in *GRASP65[LacZ]* heterozygous tissues when compared to wildtype, and appears lower in homozygous tissues (Fig. 4A). We do not understand the biological significance of these results but, overall, these data show no compensatory upregulation of GRASP55 and GM130 resulting from loss of GRASP65 expression.

### Functional read-outs of Golgi ribbon unlinking reveal specific defects at the cis Golgi upon loss of GRASP65 expression

The apparent lack of effect on Golgi morphology does not match the reported effects of GRASP65 depletion by siRNA or ablation by killer-red (Jarvela and Linstedt, 2014) that have been shown to result in Golgi ribbon unlinking, specific to the cis side of the Golgi stack (Jarvela and Linstedt, 2014) and mild glycosylation defects (Jarvela and Linstedt, 2014; Xiang et al., 2013).

To assess Golgi ribbon unlinking in a functional manner, we turned to the well established FRAP assay (Feinstein and Linstedt, 2008; Jarvela and Linstedt, 2014; Puthenveedu et al., 2006). The reasoning behind this assay is that the tubular connections between adjacent cisternae of same nature present in a Golgi ribbon would allow protein diffusion in the plane of the membrane. Photobleaching of a fluorescently tagged Golgi transmembrane protein would be followed by a quick recovery when the Golgi ribbon is intact and would be slower if the tubular connections are impaired.

**Fig. 6.**
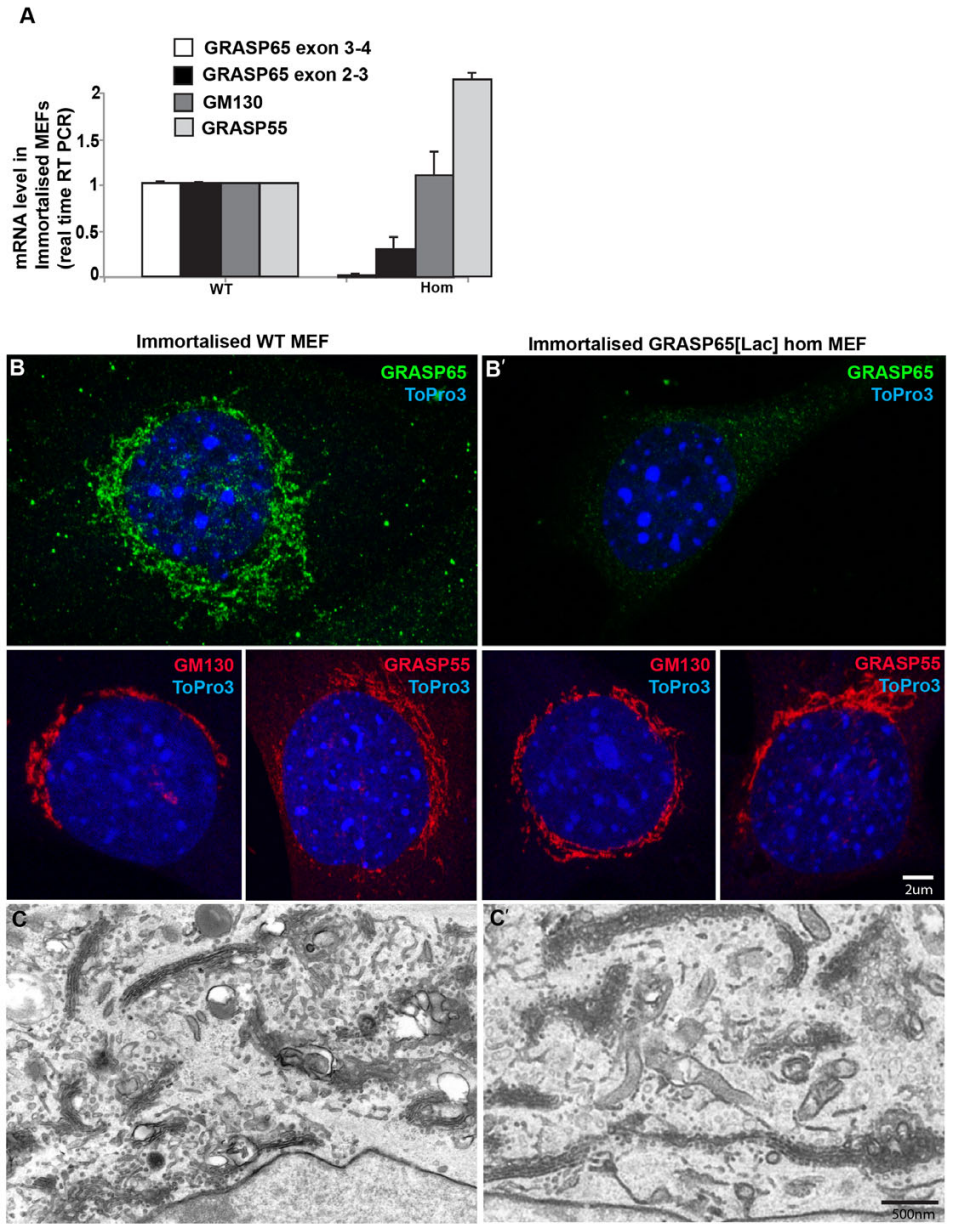
Characterisation of immortalised MEFs. (A) Measurement of *GRASP65*, *GRASP55* and *GM130* mRNAs by real time RT PCR. (B,B′) Immunofluorescence visualization of GRASP65, GRASP55 and GM130 in wild type (B) and *GRASP65[LacZ]* homozygous (B′) immortalized MEFs. (C,C′) Golgi profiles on ultrathin epon sections (flat embedding) of wild type (B) and *GRASP65[LacZ]* homozygous (B′) immortalized MEFs. Scale bars: 2 µm (B,B′), 500 nm (C,C′).

However, it is not possible to perform this assay in tissues, so we derived primary mouse epithelial fibroblasts (MEFs) (supplementary material Fig. S4A,B) and immortalized them by PSv7 transfection (Fig. 6). Their characterization reveals that *GRASP65* mRNA is absent, whereas *GRASP55* mRNA is at wild type level and *GM130* mRNA is expressed 2-fold (Fig. 6A). This was not matched at the protein level (Fig. 6B). Quantitation using image J of the immunofluorescence labeling of GM130 at the Golgi where it exclusively resides shows that the level is 25±7.5% reduced in the mutant MEFs. Last, we assessed the architecture of the Golgi ribbon both in primary (supplementary material Fig. S4C,C′) and immortalized (Fig. 6C) wild type and *GRASP65[LacZ]* MEFs. As in tissues, we did not observe differences in Golgi organization.

**Fig. 7.**
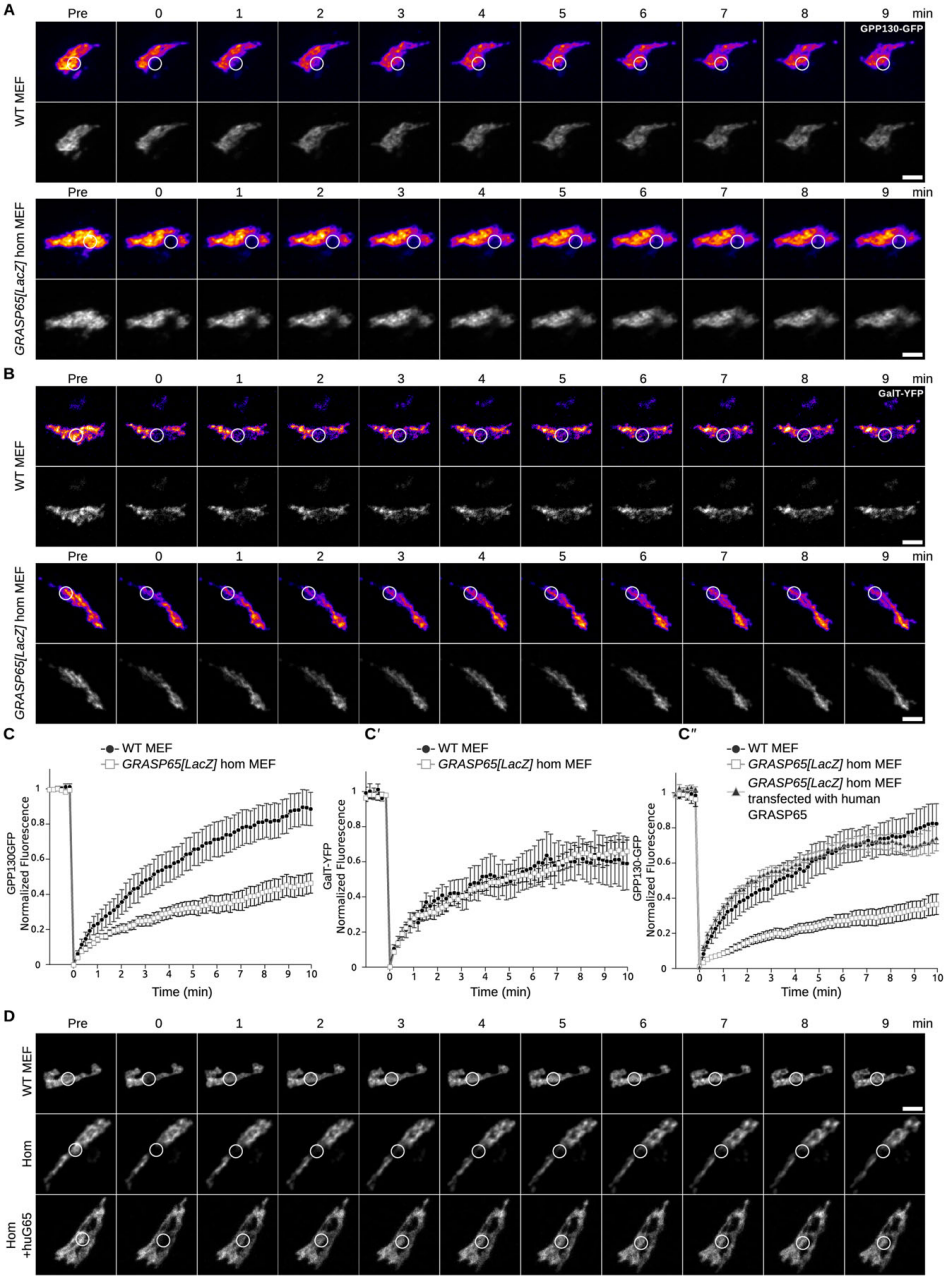
FRAP on immortalised MEFs reveals specific cis Golgi unlinking. (A) Time course of Golgi fluorescence recovery after photo-bleaching of GPP130-GFP in wild type (WT) and *GRASP65[LacZ]* homozygous (Hom) MEFs. White circles encompass the photo-bleached region. The top row shows a heat map of intensity from yellow (strong) to blue (weak). (B) Time course of Golgi fluorescence recovery after photo-bleaching of GalT-YFP in wild type (WT) and *GRASP65[LacZ]* homozygous (Hom) MEFs. White circles encompass the photobleached region. (C–C″) Fluorescence levels in bleached region is measured and is plotted versus time (*n* = 10 cells, mean ± s.e.m., *P<0.05, n.s.  =  not significant). Panel C corresponds to panel A, panel C′ to panel B, and panel C″ to panel D. (D) Time course of Golgi fluorescence recovery after photo bleaching of GPP130-GFP in wild type (WT) and *GRASP65[LacZ]* homozygous (Hom) MEFs as well as Hom MEFs back transfected with human GRASP65. White circles encompass the photobleached region. Scale bars: 5 µm (A,B,D).

To monitor Golgi ribbon unlinking in a functional manner by FRAP (see above), we transfected wildtype and *GRASP65[LacZ]* immortalized MEFs with two types of Golgi markers, the cis Golgi protein GPP130-GFP and the trans Golgi enzyme GalT-GFP (Feinstein and Linstedt, 2008; Jarvela and Linstedt, 2014; Puthenveedu et al., 2006). The recovery of photobleached GPP130-GFP in wildtype MEFs is very quick (Fig. 7A,C) and significantly reduced (50–60%) in *GRASP65[LacZ]* MEFS (Fig. 7B,C′). The recovery is similar to wildtype level upon re-introduction of human GRASP65 in the *GRASP65[LacZ]* MEFs (Fig. 7D,C″). This shows that in GRASP65 loss of function, the Golgi is functionally unlinked, at least at the cis side. Conversely, the recovery of photobleached GalT-GFP was unaffected in *GRASP65[LacZ]* MEFS when compared to wildtype, suggesting that the Golgi ribbon is unaffected at the trans side and that unlinking is specific to the cis side.

### Golgi ribbon unlinking does lead to glycosylation defects

**Fig. 8.**
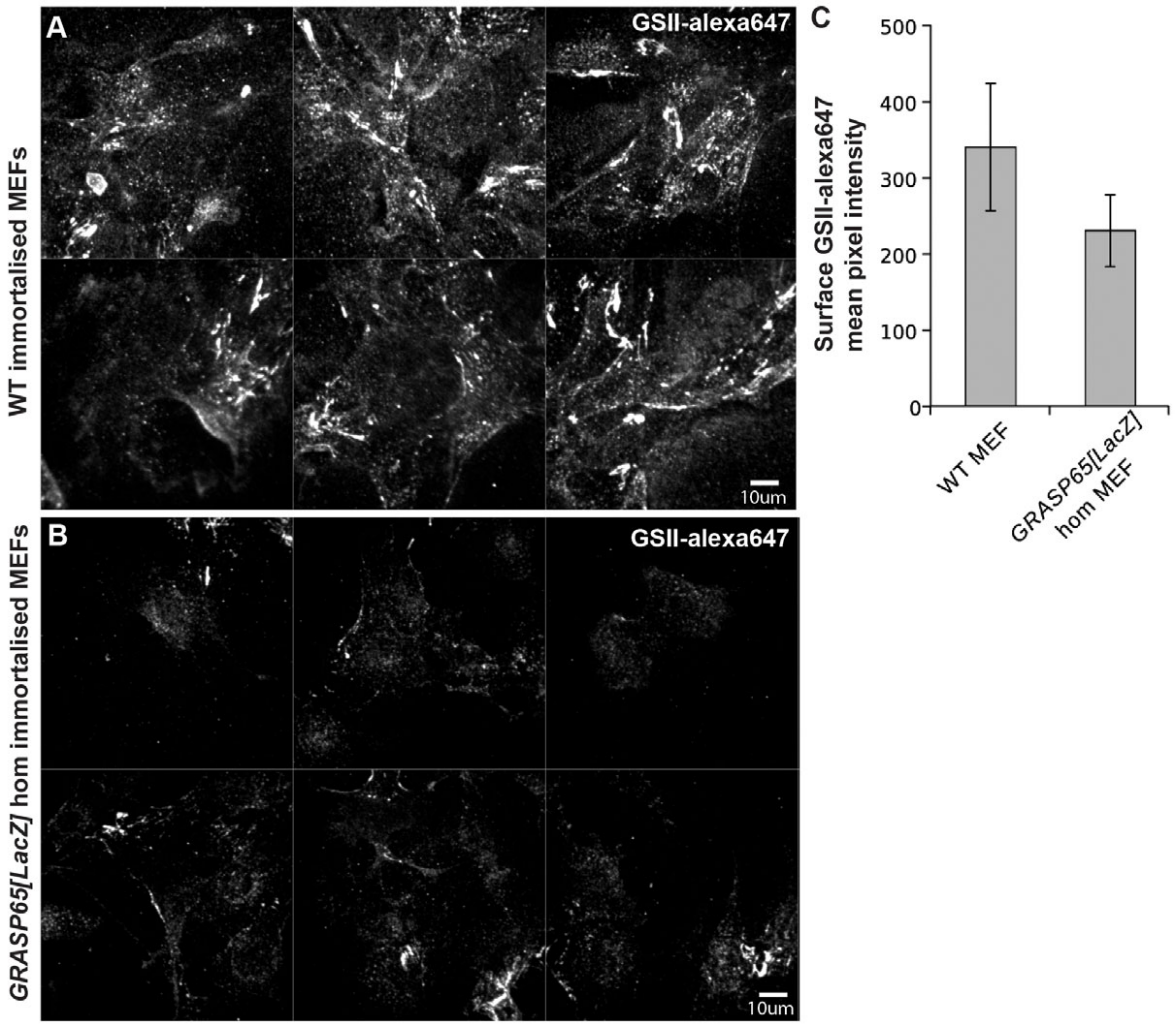
Protein N-linked glycosylation is affected in *GRASP65[LacZ]* homozygous MEFs. (A,B) GSII-alexa647 lectin staining of the plasma membrane of wildtype (A) and homozygous *GRASP65[lacZ]* (B) immortalized MEFs. Note that it is strongly decreased in the homozygous MEFs. (C) Quantitation of the fluorescence level as a surface mean intensity (*n* = 38 WT and 41 hom MEFs). Scale bars: 10 µm (A,B).

Golgi ribbon integrity has been shown to be important for proper Golgi glycosylation as it allows for an even distribution of Golgi resident enzymes across the entire organelle. As the loss of GRASP65 leads to Golgi ribbon unlinking at the cis side, we tested whether this led to a change in the distribution and intensity of the GSII lectin. This lectin detects terminal N-acetyl-D-glucosamine present in oligosaccharide chains in which the addition of this sugar moiety has been catalyzed by NAGT1 and 2 in the medial Golgi (Rabouille et al., 1995). Surface staining of GSII is significantly lower in the *GRASP65[LacZ]* MEFs when compared to WT (Fig. 8A,B), suggesting a strong change in the N-linked glycosylation pattern, resulting from the cis Golgi unlinking.

**Fig. 9.**
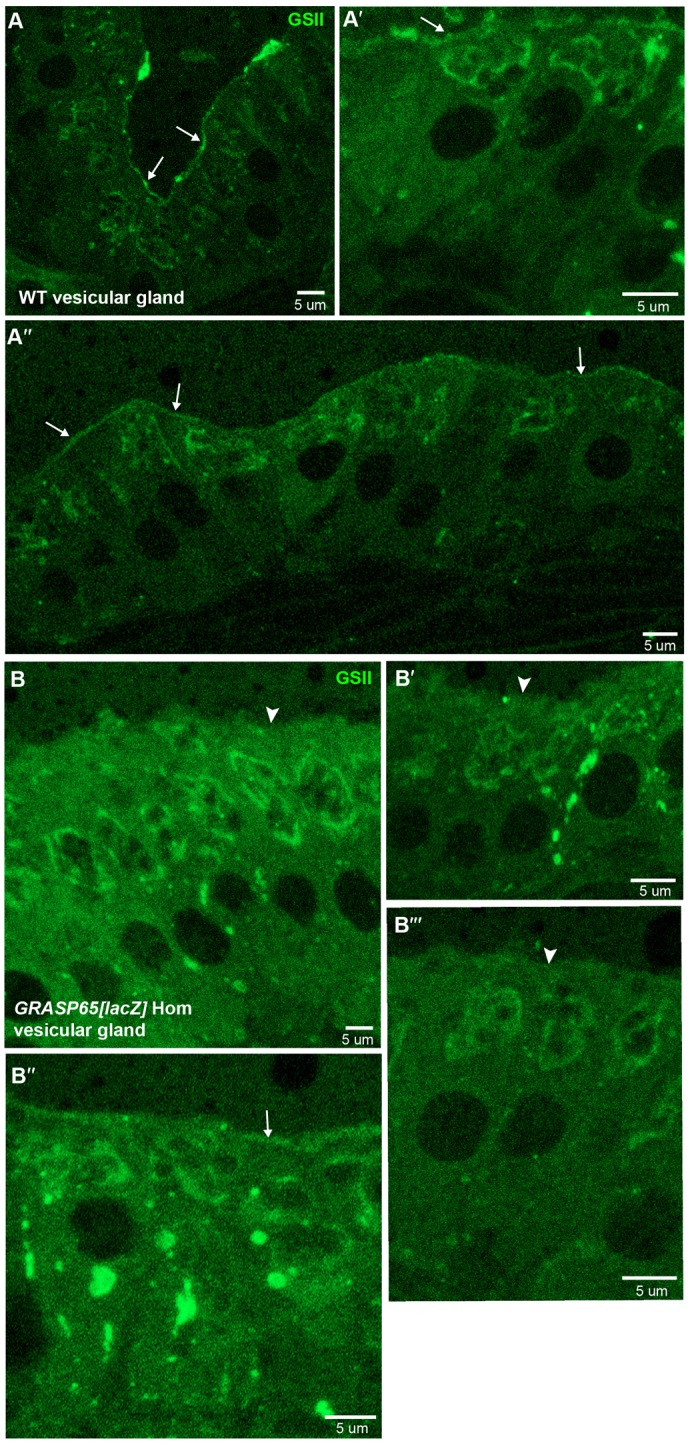
Protein N-linked glycosylation is affected in *GRASP65[LacZ]* homozygous vesicular glands. GSII-alexa647 lectin staining of frozen thin sections of wild type (A–A″) and homozygous *GRASP65[lacZ]* (B–B‴) vesicular glands. Note that the plasma membrane staining is strongly decreased in the homozygous tissues whereas the Golgi staining increases together with intracellular dots. Arrows indicates GSII plasma membrane staining and arrowheads, absence of GSII staining. Scale bars: 5 µm.

Last, we assessed GSII staining on tissues using thin frozen sections of vesicular glands and pancreas of wild type and *GRASP65[LacZ]* mice. In wild type vesicular gland, GSII stains the Golgi and the plasma membrane, as expected whereas in the mutant tissue, the plasma membrane staining is decreased or gone and the Golgi staining seems to increase (Fig. 9).

The change at the cell plasma membrane in the vesicular gland recapitulated well what we observed at the MEF's plasma membrane. Note that the Golgi in MEFs would not be assessed because staining was extracellular to exclusively detect sugars exposed in plasma membrane proteins, whereas on thin sections, as those presented, the intracellular space is accessible to the lectin.

In wild type pancreas (supplementary material Fig. S5), the plasma membrane of the duct was labeled with GSII (as well as at the Golgi). In the mutant tissue, the duct staining was not decreased as we observed in the vesicular gland, suggesting that the glycosylation defects might be tissue specific.

## DISCUSSION

Here, we show that the GRASP65 gene disruption by targeted gene knock-in (*GRASP65[Lacz]*) in mice does not lead to developmental phenotype and adult homeostasis defects. The homozygous *GRASP65[LacZ]* mice are viable and fertile and their longevity so far is identical to their wild type littermates when raised in standard laboratory conditions. Accordingly, we could not detect tissues defects and primary and immortalized MEFs have similar growth as their wild type counterpart.

This result is a little unexpected given the number of roles attributed to GRASP65 (reviewed by Vinke et al., 2011), in particular in mitosis and apoptosis. At least 4 serine/threonine residues (Ser216/Ser217, Thr220, Ser277 and Ser376) in C-terminus of GRASP65 have been identified as target by the key mitotic kinase CDK1 (cyclin-dependent kinase 1) (Preisinger et al., 2005). In turn, GRASP65 mitotic phosphorylation leads to the loss of the Golgi-stacked architecture (either Golgi ribbon unlinking and/or Golgi fragmentation). This can be explained by the fact that the phosphorylation of these residues has been shown to lead to impairment of GRASP65 dimers to form higher order oligomers (Wang et al., 2005; Wang et al., 2003) and therefore a loss of the tethering properties.

Remarkably, interfering with GRASP65 has been shown to result in defect in mitotic entry. The first observation was made using microinjection of an inhibitory antibody targeting part of the C-terminal region of GRASP65 that led to the inhibition of G2 Golgi fragmentation (as assessed by immunofluorescence microscopy), as well as the failure of cells to proceed through mitosis (Sütterlin et al., 2002). Furthermore, preventing GRASP65 phosphorylation, thus preventing Golgi ribbon unlinking, also results in a delay in mitotic entry (Tang et al., 2010).

However, GRASP65 depletion by RNAi does not prevent mitotic entry. GRASP65 depletion would result in Golgi ribbon unlinking (at least at the cis side), which would allow the cells to pass through the G2/M checkpoint (Sütterlin et al., 2005). Therefore, in agreement with the cell culture result, loss of GRASP65 expression in the *GRASP65[LacZ]* mice we generated should not impair this checkpoint either.

GRASP65-depleted HeLa cells have also been reported to display multiple, disorganized and non-functional spindle poles and their chromosomes do not align properly on the metaphase plate. As a result, GRASP65-depleted cells do pass the metaphase checkpoint, exit mitosis, and quickly undergo apoptosis (Sütterlin et al., 2005). Our results are contrasting with this observation. Such defects were not seen in the *GRASP65[LacZ]* knock-in MEFs or tissues. We have not studied in detail the centrosome organization, but we do not observe developmental defects and MEF survival and proliferation. We therefore strongly suspect that the mitotic program is fine.

The apoptotic caspase 3 has been shown to cleave GRASP65 at three specific sites present in its C-terminus (Asp320, Asp375 and Asp393) and expression of a caspase-resistant form of GRASP65 inhibited Golgi fragmentation (Lane et al., 2002). As for mitosis, it seems that the expression of the resistant form is more detrimental than the absence of the protein. It is likely that loss of GRASP65 protein would not impair apoptosis progression as, in its absence, many other Golgi substrates might take over and leads to Golgi fragmentation.

Taken together, it is clear that the absence of the GRASP65 protein is less deleterious than the presence of a form that cannot be phosphorylated (mitosis) or is caspase resistant (apoptosis). Overall, except for the spindle assembly and the centrosome organization, our results do not disagree with a role for GRASP65 in mitotic progression or in driving apoptosis. In this regard, it would be interesting to replace endogenous GRASP65 by mutated forms and then assess the phenotypes.

Mammalian cells have a second GRASP (GRASP55) that we find is much more abundant than GRASP65, at least at the transcriptional level, although it is not upregulated in the absence of GRASP65. It is therefore possible that either GRASP55 compensates the loss of GRASP65 by taking some of its function, or that the loss of GRASP65, given its low level of expression is not enough to lead to measurable defects at the tissues and organismal level. In this regard, it will be important to examine the developmental and adult defects in a GRASP55 loss of function mammal.

Upon examination of the Golgi structure in tissue or MEFs at the (sub)cellular level and ultrastructural level, the loss of GRASP65 dis not lead to visible structural alterations, either in term of cisternal stacking or Golgi ribbon integrity. Surprisingly, functional assessment of the Golgi ribbon integrity by FRAP in immortalized MEFs reveals strong defects in the ribbon integrity at the cis, but not trans, side of the Golgi complex of the *GRASP65[LacZ]* MEFs. We therefore confirm here the extensive evidence that GRASP65 that has a localization biased to cis cisternae (Shorter and Warren, 2002) plays a direct role in cisternae-specific tethering in cultured cells.

Thus, our findings indicate that GRASP65 plays a role in sustaining cis cisternae linkages in total agreement with depletion of GRASP65 by siRNA in HeLa cells or killer-red technology, suggesting a very strong homology between HeLa cells and MEFs. Therefore, whether the loss of GRASP65 is acute or long term, the effect of losing GRASP65 is the same in all the cell lines used, although, surprisingly, it is only revealed by performing FRAP functional assay in MEFs, as the Golgi morphology (by light and electron microscopy) was not enough to reveal defects, whether on tissues or in MEFs. This result, however, is in agreement with the specific (therefore subtle) unlinking at the cis side.

The loss of Golgi integrity in *GRASP65[LacZ]* leads to glycosylation defects as shown by a strong decrease in N-acetyl-D-glucosamine pattern assessed by Alexa-647 conjugated GSII lectin when compared to wild type. This difference is, however, reverse from this observed in HeLa cells depleted from GRASP65 (Puthenveedu et al., 2006; Jarvela and Linstedt, 2014) in which the GSII staining is higher when compared to control cells. We argue that this difference might be due to the fine Golgi (sub)localization of the enzymes catalyzing the addition of N-acetylglucosamine on N-linked oligosaccharides borne by proteins in transit through the Golgi en route to the plasma membrane (NAGT1 and 2). The observed GSII staining is consistent with a localization of these enzymes in the cis/medial cisternae in HeLa cells (Rabouille et al., 1995) whereas they would be localized more to the medial/trans in the MEFs. In such a scenario, the cis Golgi unlinking would not allow anterograde cargo to acquire terminal N-acetylglucosamine, leading thus in a decrease of plasma membrane staining. This was recapitulated in the vesicular gland but not in exocrine pancreatic ducts, indicating that the glycosylation defects due to loss of GRASP65 loss of expression are tissue specific. Furthermore, these glycosylation defects do not lead to a developmental or adult phenotype, at least in standard laboratory conditions.

Whether stress conditions would reveal organismal phenotypes remains to be explored. It will also be critical to examine the phenotype of a GRASP55 loss of expression mouse as well as the double mutant.

## MATERIALS AND METHODS

### Generation of *GRASP65[LacZ]* knock-in mouse

*GRASP65[LacZ]* knock-in mice were generated by homologous recombination in embryonic stem cells using a targeting construct R3R4_pBR_DTA+_Bsd_amp (from KOMP, https://www.komp.org) that is schematically depicted in Fig. 1A and the full sequence is in supplementary material Fig. S1. It contains the GRASP65/GORASP1^tm1a(KOMP)Wtsi^ allele as well as neomycin resistance, Lacz and two LoxP sites.

100 µg of targeting construct was linearized and transfected into male 129/Ola-derived IB10 embryonic stem (ES) cells by electroporation (800 V, 3 F) that are maintained undifferentiated over a feeder layer of MEFs in the presence of leukaemia inhibitory factor (LIF). Recombinant ES cell clones that are resistant to neomycin were selected in medium supplemented with G418 (250 µg/ml). Approximately 300 recombinant clones were screened by Southern blotting using a PCR generated 3′ flanking probe in order to confirm the site-specific integration of the LoxP sites (Fig. 1A). The sequence and positions of primers used are indicated in Fig. 1A and Table 1. About 9% of the isolated clones showed homologous recombination (Fig. 1B).

The targeted ES cells from two independent clones were injected into blastocysts derived from C57BL/6 mice and implanted into pseudo-pregnant female ICR mice using standard techniques.

Resulting GRASP65 chimeric animals (showing patches of agouti color fur into the otherwise black fur) were backcrossed to C57BL/6 to produce heterozygous mice carrying one wildtype allele and one allele in which the GRASP65 genomic locus has been recombined to the targeting vector (*GRASP65[LacZ]*). Because of the nature of the targeting vector, the *GRASP65[LacZ]* knock-in mouse have lost the GRASPP65 genomic locus expression. This in animals was confirmed by PCR and RT-PCR on tail (Fig. 1C) and RT-PCR on kidney, vesicular gland and MEF tissue (Figs 3 and 6) (Galli-Taliadoros et al., 1995; Kühn and Schwenk, 1997; Mantamadiotis et al., 1998; Müller, 1999; Skarnes et al., 2011).

Mice used in experiments were sex and age matched and kept in pathogen free conditions. The animal experiments were approved by the Animal Experimentation Committee of the Royal Academy of Arts and Sciences (protocol number HI 12.1004).

### Quantitative RT PCR measurements of mRNA levels

RNA extraction from kidney, vesicular glands and MEFs was performed using Trizol® (Life Technologies, 15596018). cDNA was synthesized using the Goscript Reverse Transcriptase (Promega, A5000) based on 1000 ng of total RNA per 15 µl reaction.

qPCR was performed on the resultant cDNA using IQ Sybr Green Supermix (BioRad, 170-8887), with probes for mouse Gapdh1 (NM_008084.2), *GRASP65* (NM_0289762), *GRASP55* (NM_027352.4) and B2M (NM_009735.3).

The levels of *GRASP65*, *GM130* and *GRASP55* mRNAs were normalized to the expression of housekeeping B2M and Gapdh1 mRNAs. *GRASP65*, *GM130* and *GRASP55* mRNA levels were expressed as a fraction of the wildtype sample.

### X-Gal (5-bromo-4-chloro-3-indolyl-β-d-galactopyranoside) staining

To determine the pattern of the *GRASP65[LacZ]* reporter locus in mice, tissues from were fixed for 2 h in cold fixative (1% Formaldehyde (Sigma, F8775); 0.2% Glutaraldehyde (Merck, 104239); 0.02% NP-40 in PBS) and incubated overnight at room temperature with 1 mg/ml of X-gal (bromo-chloro-indolyl-galactopyranoside, Life Technologies, 15520-018) solution as we described (Barker et al., 2007). The stained tissues were transferred to tissue cassettes and paraffin blocks were prepared using standard methods. Tissue sections (4 µm) were prepared and counterstained with neutral red marking the nucleus and sometimes the cytoplasm at a lower level. Pictures were taken with a Nikon E600 camera.

### Antibodies

Rabbit anti rat GRASP65: bacterially expressed rat N-terminally tagged GST-GRASP65 (from a pGEX vector) was injected in rabbit. The serum was collected and tested in IF (1/750) and WB (1/1000) on several cell and tissue samples (Fig. 5).

Sheep anti Grasp55 (A. Linstedt, 1/4000 WB and 1/500 IF); Mouse anti rat GM130 (BD 610822, 1/1000 WB, 1/250 IF); Mouse anti sea urchin tubulin (Sigma T5168, 1/10,000 WB; Mouse anti Actin (AC-15, Santa Cruz, 69879, 1/5000 WB); Rabbit anti sheep IgG-HRP (Dako P0163, 1:2000 WB); Sheep anti Mouse IgG HRP (GE Healthcare NA931, 1/2000 WB); Donkey anti Rabbit IgG HRP (GE Healthcare NA934, 1/2000 WB); Donkey anti Rabbit Alexa fluor 488 (Life Technologies A21206, 1/250 IF); Donkey anti mouse Alexa fluor 568 (Life Technologies A10037, 1/250 IF); Donkey anti Sheep Alexa 568 (Life Technologies A21099, 1/250 IF); Phalloidin 647 (Life Technologies A2228; 1/250); Topro 648 (Life Technologies T3605; 1/500).

### Immunofluorescence microscopy

Cells were first washed with PBS and subsequently fixed with 4% paraformaldehyde (Sigma, 441244) on ice for 20 minutes. After fixation cells were quenched with 50 mM NH_4_Cl and subsequently permeabilized by 0.1% Triton X-100 in PBS. Blocking of the cells was carried out in PBS in the presence of 1% cold Fish Skin Gelatin (FSG) for 20 min. Primary antibodies were diluted 1% FSG in PBS for 30 min. Secondary antibodies (see above) were then incubated in 1% FSG in PBS and cells were washed three times with water and mounted in Prolong® (Invitrogen, P36930) for imaging on a confocal microscopy system (SPElive; Leica) at room temperature (63× NA 1.4 objective) using LAS software (Leica) for acquisition.

### Electron microscopy

Small blocks of tissues were fixed in Karnovsky's fixative (2% paraformaldehyde, 2.5% glutaraldehyde, 0.1 M sodium cacodylate (BzDH, 30118), 2.5 mM CaCl_2_, 5 mM MgCl_2_, pH 7.4) overnight at 4°C. MEFs cells were fixed in Karnovsky's fixative using a flat embedding technique in which instead of propylene oxide we used 100% ethanol dehydrated with acidified 2,2-DMP (dimethoxypropane). All samples were further processed to Epon resin using standard electron microscopy procedures and examined either with a Jeol 1200 EX or a Fei Tecnai 12 electron microscope.

### SDS-PAGE and Western blotting

Samples were run on 10% polyacrylamide minigels for 90 minutes at 150 V at RT. Proteins were transferred by wet blotting for 45 minutes at 300 A to PVDF membranes, blocked in 1× PBS in 0.05% Tween20 supplemented with 5% powdered milk (Campina, NL). Primary antibody incubations were for performed overnight at 4°C. PVDF and nitrocellulose membranes were then washed with PBS/Tween20 three times before a one hour incubation at room temperature with relevant HRP-conjugated secondary antibodies (GE Healthcare) After 6 washes with PBS/Tween20, PVDF membranes were visualized by Clarity Western ECL Substrate (BioRad, 170-5061) using Kodak medical x-ray film.

### MEF isolation and immortalization

MEFs were isolated from parental C57BL/6 (WT) and *GRASP65[LacZ]* mice. Pregnant females were sacrificed by cervical dislocation at day 13.5 of gestation, and embryonic fibroblasts were prepared by standard procedures (Conner, 2001) using TrypLE™. Primary mouse fibroblasts were expanded for 24 h in DMEM^−−^ supplemented with 10% fetal FBS (Sigma), 1% penicillin–streptomycin, later revered to as DMEM^++^ (all medium components from Life Technologies unless stated differently).

Primary MEFs P2 were seeded in a 12 well plate in DMEM^++^ (Life Technologies). Cells were immortalized by transfection with 2 µg pSV7 (gift from F. Reggiori) using Lipofectamine 2000™ (Life Technologies). The cells were incubated with the transfection medium for 4 h, washed and further incubated with DMEM^++^. The line was established after P10 and cryopreserved in 50% DMEM (Life Technologies), 40% FCS (Sigma) and 10% DMSO (Merck Millipore).

### FRAP assay

For fluorescence recovery after photobleaching, WT and homozygous immortalized MEFs were transfected with GPP130-GFP and GalT-YFP for 2 days before part of the Golgi was bleached using a single laser pulse. Images were acquired every 3 s. FRAP was measured using FRAP profiler on average projections of confocal stacks, using the freehand selection tool to select the bleached area and normalized to total cellular fluorescence. FRAP experiments were acquired with Andor iQ2 spinning-disk confocal system at the Molecular Biosensors and Imaging Center at Carnegie Mellon University (Jarvela and Linstedt, 2014).

### Lectin binding and analysis

Surface staining with Alexa 647-labeled GSII-lectin (Life Technologies) was performed after washing cells 5× in ice-cold PBS. MEFs were incubated with 5 µg of GSII lectin/ml in ice-cold PBS containing 0.5 mM MgCl_2_ and 1 mM CaCl_2_ and 1% bovine serum albumin for 20 min at 4°C. Cells were then washed 5× with the same buffer and fixed and viewed with the spinning-disk confocal system at the Molecular Biosensors and Imaging Center at Carnegie Mellon University. A fixed threshold was used to quantify the surface signals (Jarvela and Linstedt, 2014).

GSII staining on 250 nm frozen sections of chemically fixed tissue sections (as for EM) was performed using 20 µg/ml in PBS supplemented 1 mM CaCl_2_. Pictures were taken on SPElive from Leica at room temperature.

### Statistical analysis

The statistical significance of all comparisons was assessed by two-tailed Student's *t* tests, and, where indicated, non-overlap of curves was estimated using root mean squared deviation.

The immunofluorescence GM130 labeling was measured by image J by capturing the total area of labeling. 25 cells (from 3 different experiments) of each phenotype were analysed.

## Supplementary Material

Supplementary Material
