# Supplementary material for: GRASP65 controls the cis Golgi integrity in vivo
Source: Biol Open. 2014 May 2;3(6):431–43. doi: 10.1242/bio.20147757 (PMC4058077; doi:10.1242/bio.20147757)
Supplement: Supplementary Material [file supp_3_6_431__index.html]

GRASP65 controls the cis Golgi integrity in vivo — Supplementary Material 

# GRASP65 controls the cis Golgi integrity in vivo

## bio.20147757 Supplementary Material

**Files in this Data Supplement:**

- Supplementary Material - Tineke Veenendaal et al. doi: 10.1242/bio.20147757
- Figure S1 - **Information related to Fig. 1A.** (A) Position and symbol of key element in the targeting vector. (B) Full sequence of the annotated targeted GRASP65 gene.
