# Supplementary material for: GRASP65 controls the cis Golgi integrity in vivo
Source: Biol Open. 2014 May 2;3(6):431–43. doi: 10.1242/bio.20147757 (PMC4058077; doi:10.1242/bio.20147757)
Supplement: Supplementary Material [file supp_bio.20147757_bio.20147757-s1.pdf]

Biology Open (2014) 000, 1–13 doi:10.1242/bio.20147757

Supplementary Material
Tineke Veenendaal et al. doi: 10.1242/bio.20147757

Fig. S1. Information related to Fig. 1A. (A) Position and symbol of key element in the targeting vector. (B) Full sequence of the annotated targeted GRASP65 gene. See supplementary material web page for this figure.

Fig. S2. Matching GRASP65 exons 1–3 to protein sequence. (A) *GRASP65* genomic region around exons 1–3 matching DNA sequence to protein sequence. (B) Exons 1–3 encodes GRASP65 PDZ1 (according to Truschel et al., 2011).

A

mus musculus GRASP65 genomic sequence (till exon 3)

5′UTR
1 ccccacgtgactaggccacgcagcagcgggagagggcggcc

Exon 1
42 atggggctaggggcaagcagcgagcagccggcgggcggcgagggcttccatctgcacggggtg

>>1 MGLGASSEQPAGGEGFHLHGV

Intron 1: 104-4219

Exon 2
4220 caagagaactcgccggcccagcaggcaggcctggagccctacttcgacttcatcatcaccatcgggc
actcgaggctggtg

>>22 QENSPAQQAGLEPYFDFIITIGHSRL

Intron 2: 4301-4734

Exon 3
4735 aacaaggagaacgacacgctgaaggcattgctgaaggccaatgtggagaagccggtgaagctggag
gtattcaacatgaagaccatgaaggtgcgcgaggtagaggtggtgcccagcaacatgtggggcggccagg
gcctcctgggagccagcgtgcgcttctgtagcttccgcagggccagcgaacacgtgtggcatgtgctg

>>48 NKENDTLKALLKANVEKPVKLEVFNMKTMKVREVEVVPSNMWGGQGLLGASVRFCSFRRASEHVWHVL

B

GRASP65 PDZ1 expression after KI of *GRASP65[LacZ]*. The ligand-binding domain formed by the second α-helix (shown in blue) and second β-strand (shown in red) is still present (according to Truschel et al, 2011)

1
MGLGASSEQPAGG**EGFHLH**GVQENS**PAQQA**GLEPYF**DFIITI**GHSRLNKE**NDTLKALLKAN**VEKP**VKLEVFN**MK
beta3 alpha1 beta4 alpha2 beta6

115
TMKVREVEVVPSNMWGGQGLLG**ASVRFCS**FRRASEHVWHVL
beta1 beta2

Biology Open

Biology Open (2014) 000, 1–13 doi:10.1242/bio.20147757

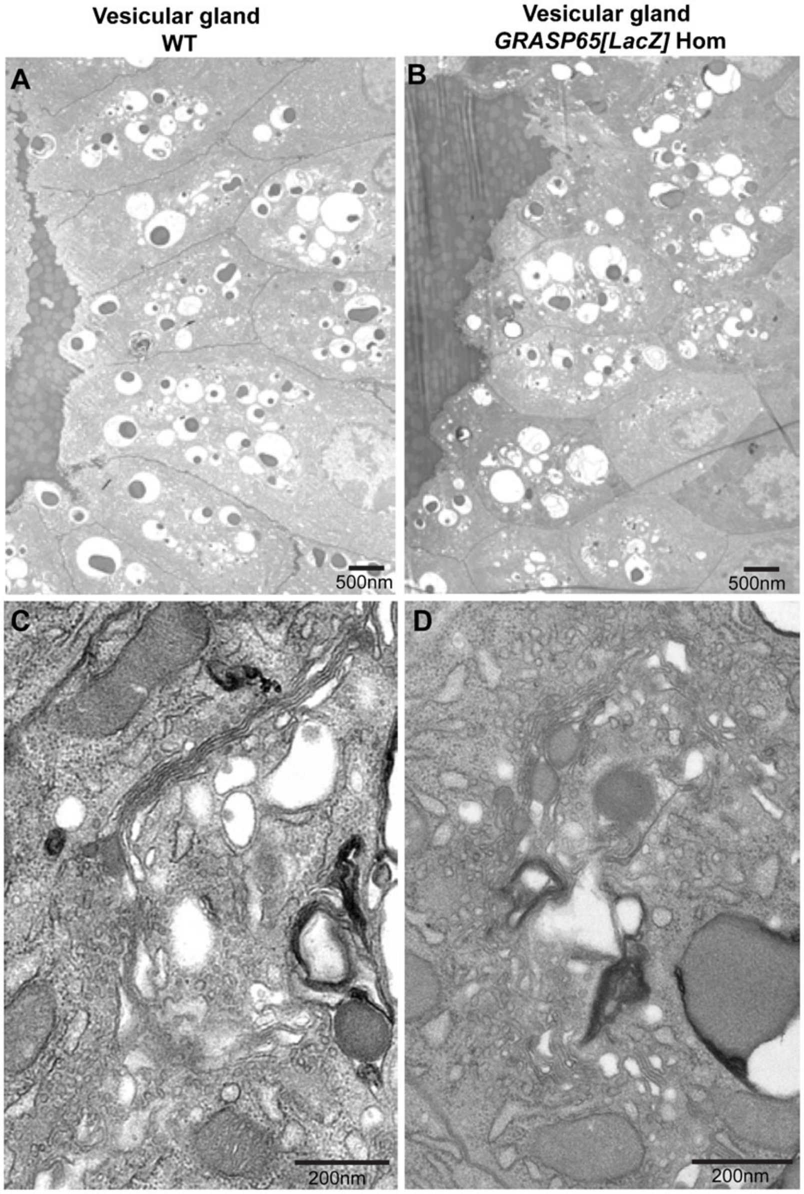

Fig. S3. The Golgi apparatus appears normal in KO vesicular glands. (A,B) Low magnification of vesicular glands ultrathin epon sections from WT (A) and *GRASP65[LacZ]* hom (B) mice. (C,D) Golgi profiles in ultrathin epon sections of vesicular glands from WT (A), het (C) and *GRASP65[LacZ]* hom (D,F) mice. Note that no differences are detectable. Scale bars: 500 nm (A,B), 200 nm (C,D).

Biology Open (2014) 000, 1–13 doi:10.1242/bio.20147757

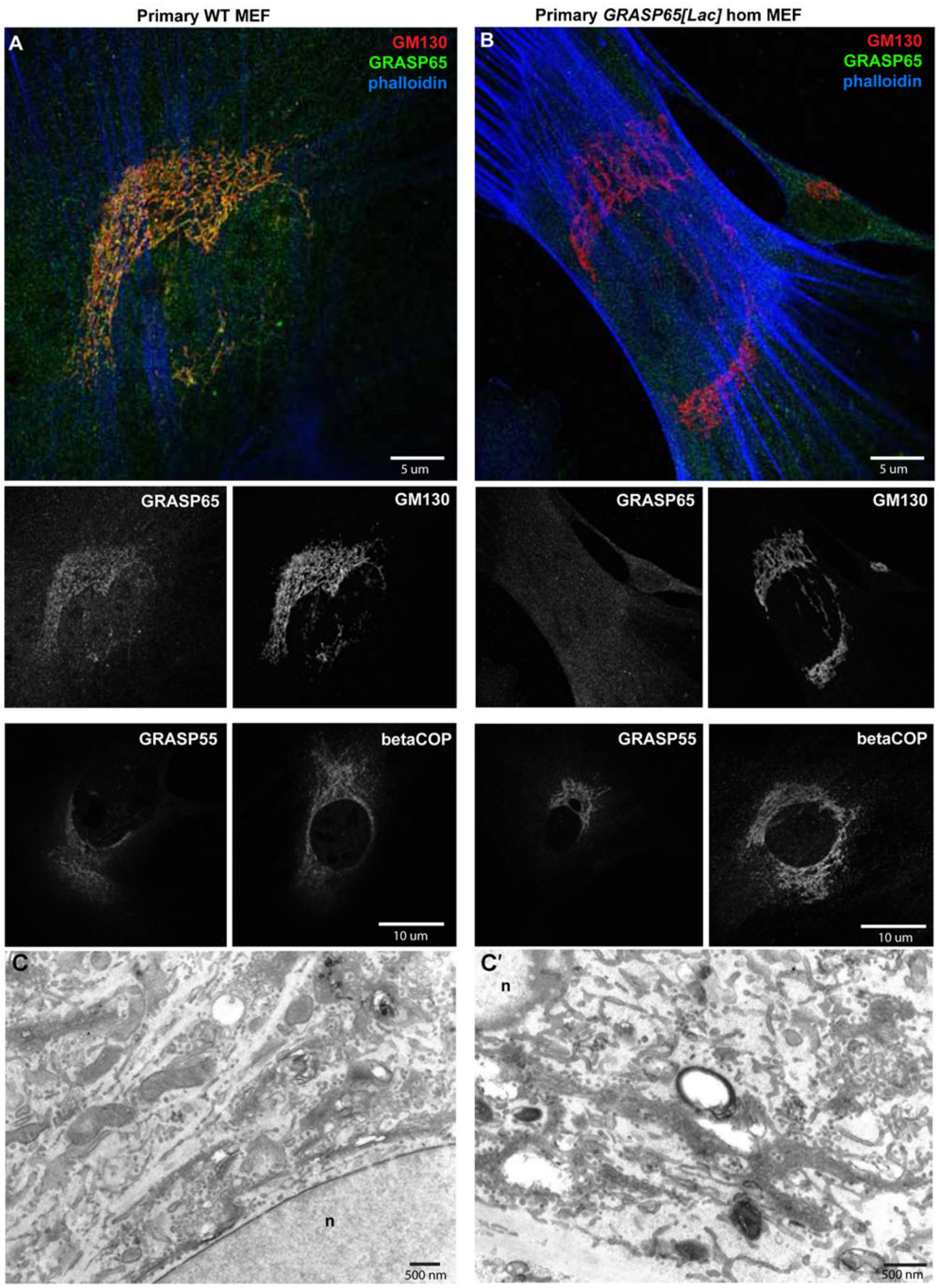

Fig. S4. Characterisation of the primary MEFs. (A,B) Immunofluorescence visualisation of GRASP65, GRASP55, GM130 and betaCOP in primary WT (A) and *GRASP65[lacZ]* homozygous (B) MEFs. (C,C′) High magnification view of Golgi profiles in ultrathin epon sections of primary WT (A) and *GRASP65[LacZ]* Hom MEFS (A′). Note that no differences are detectable. Scale bars: 5 µm (A,B), 10 µm (middle panels), 500 nm (C,C′).

Biology Open

Biology Open (2014) 000, 1–13 doi:10.1242/bio.20147757

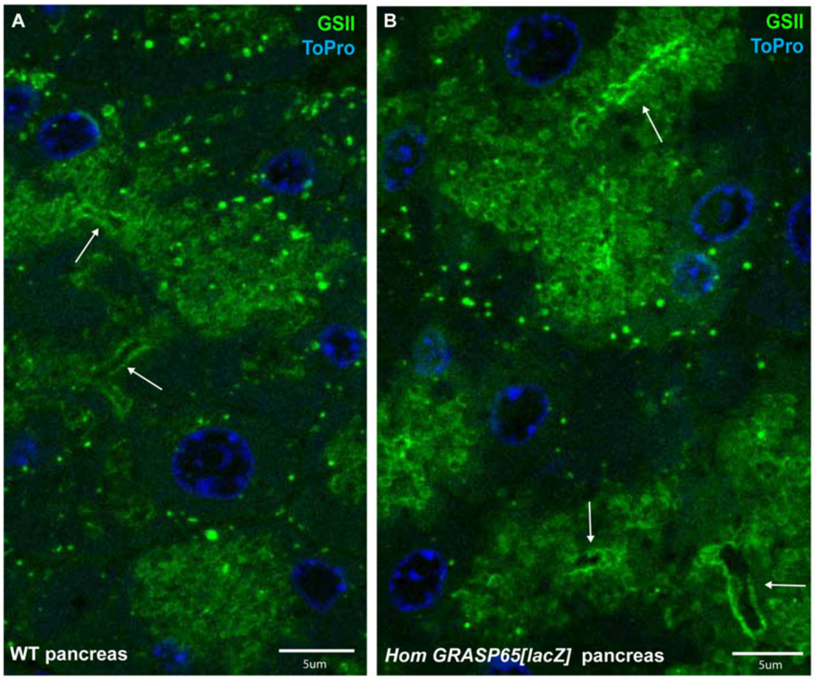

Fig. S5. GSII lectin staining of exocrine pancreas. GSII (green) and ToPro (blue, nucleus) staining of thin frozen section of fixed wild-type (A) and GRASP65[LacZ] (B) exocrine pancreas. Note that the ducts (apical plasma membrane) marked by arrows are stained in both. Scale bars: 5 μm.
