# Supplementary material for: GRASP65 controls the cis Golgi integrity in vivo
Source: Biol Open. 2014 May 2;3(6):431–43. doi: 10.1242/bio.20147757 (PMC4058077; doi:10.1242/bio.20147757)
Supplement: Supplementary Material [file supp_bio.20147757_Fig._S1.docx]

**Supplementary Figure S1: Veneendaal et al, 2014**

**A**


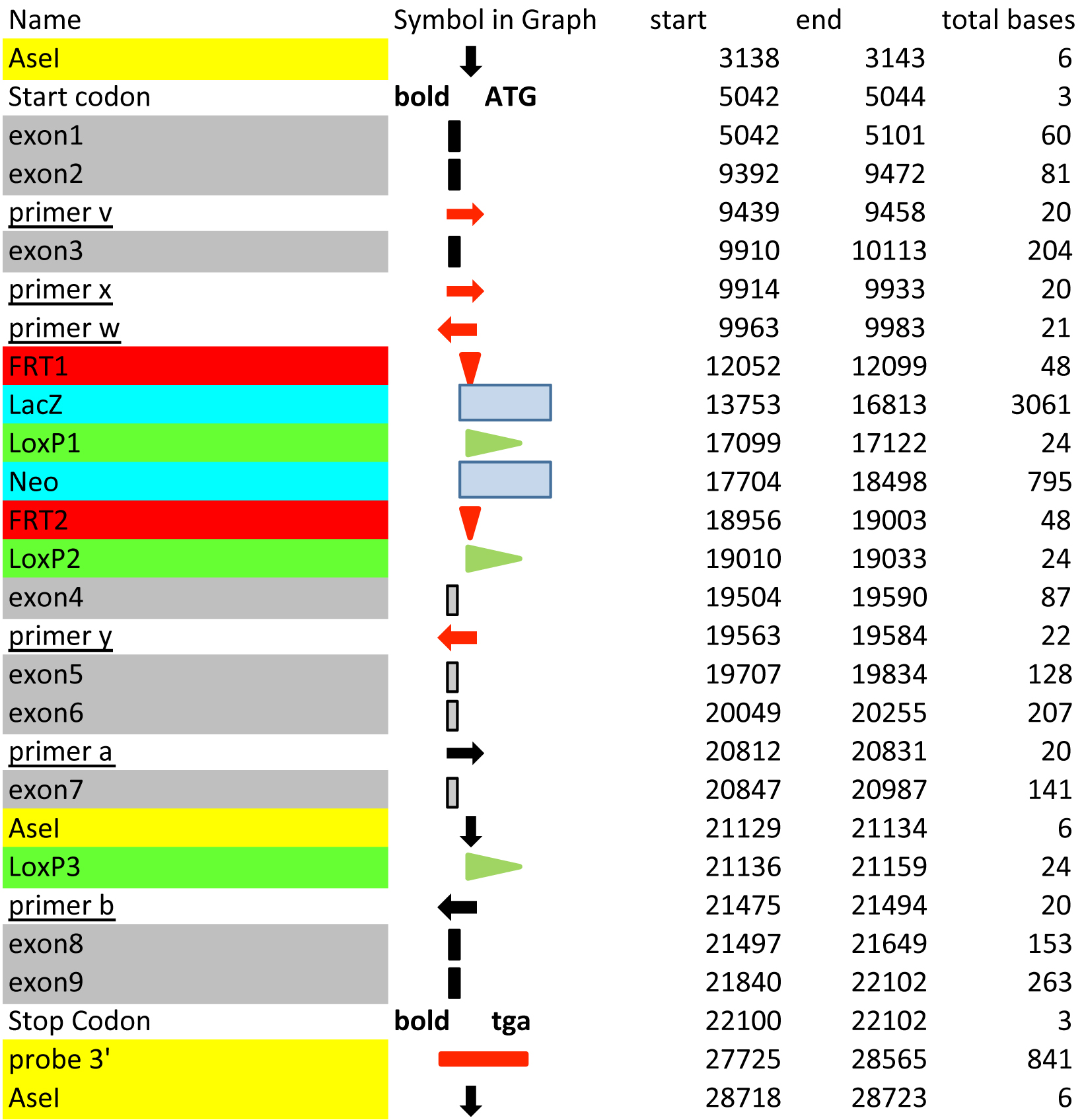


**B**

1 AAGGGGAAAGGTGAAGTTCTGCGTCCTGTCCAGCCTCTGCCCCAGCCGCTGGTGCCCTGC

61 CCTCCACCTGGTAGGCCGCATCGTCTTGTGCAGATCCCGCTGGGAGGCTCCTCTGCCAGG

121 TGGTGTCCCCTGCCTGCACTGCCTGCAACACCCTGACACCTCTTACTTTCCAGATCGGGC

181 CTCATACATCTGGTAAAGGACTTGGTTACAGCTTAGCCTGGGAACCCCCATCCCACACCC

241 AGGGGCCTGGTTTTCACGCATCTCTACCACTCTTCACAGCTCTTCATTGCTCCTTATGGA

301 GGGACGCCCTCGCCCCTCTCCCCATGTCCTGGACACAGGCAGTCAGGTTACCAGTACTGG

361 GGCTTGGGTAGGACTGCTCTTGCCCTCTGCACACCCTCTTCCAGTTACAGGGAGGGCCGG

421 GCCTGGGGAGTGCGCACCACCATATGGATCCCAGAGATCAAACTCCGGTCATCAGGCTTG

481 GCAGCAAACGCCTTGGCTCAGCAGATGCCTGAGCCATCTTGCCAGCCGGATCGTCTGGTT

541 TTCTACAGGTCAGCAACTGATTCAAGATGCTTAAAGAGTGCTGGGACGAAGCCCTGGCCT

601 GCTCCCCTAGAGGACTTTGGTTCTGTTTCCAGTACCCACGCGGAAGCTGACAAACGCCTG

661 TCACGCATGTGGTGCAGACACAACACCCATACACGTCAAAAATAAGTTCAAAACAAAAAT

721 TGAAAAAAAAAAACGTGGTACTGCAATGCATGTGTATGGGAAGCCCTGGGTTTCAGCCCT

781 GTGTGGAATAAGACAAGGCATGGGATAGCCTTAGAATCTCAGTACTCTAGAGTTGGGAGG

841 CAGGCGGATCAGAAGTTCAAGGCCACCTTTGCTACAGTGCGAACTGTCTGAGACCTTGTC

901 ATGAGATCTCATTGCAGAACCCAGGCCTTGAGTACAACAGCTGCTACCACCATTCTCAGA

961 AGCAGCTGTGGCCACCCACTCATAGGCTGGTCTCCTTGCTGTCCATCACTAGAGGAGGAG

1021 TTGGGAGGGTCATTTAGACCTGCCTGTGCCTCACTTTGGCTCACTGCCAGGCGTATGTTT

1081 TTGTCCCCACGCCCACCCCGAGACAGCATTTTTTTTTTTTTTTTTTTTTTTTTGAGACAG

1141 CATTTTTCTGTGTAGCCCTGGCTGACCTGGAACTTGCTCTATATAGACTGGCCTCAATCT

1201 CTGGGCGTGAGACCGCTATAATGCTGCTTTGCTTGGCCGCGGCTGACACCCTCCTGTACC

1261 TCTTTGGAGCCGCTGGAGACCTTCAACATGTGGTCAACATACTCCCTGGCCTTGTCGTGG

1321 TGACCCATGAACCAGAGGAAGAGGCTGGCGTAGTACAGAGCAGTGCTGCTGGCTGACCTG

1381 CGAATTTCCTTCAAGCTGCTCTCCAGTTCCTGAACTGCTTCTATGTCTACATCGAGGAGG

1441 AGGAGGAGGAGGAGGAGTGAGGAGTGAGGAGCGGATGTCCTCTTCTGATGCATTGTACCC

1501 TCTCTGCACCAGCGACGCTGCCCACATCCTTGGAGTTGTGTATACTTGAGCGCAGTTTAC

1561 TGTCCGGGAGGCCCCACCCCACCCCATGTCCACCAGGGTGTCAAGCAGACGGACTAACCC

1621 TGAGTTCCTGACTGAGGCTCACAGTCCATCCTGAGTGAGAGATGGAAGTCTCCACTAGCA

1681 CCTCTCTCTGACTCCCTAGAGACCTCAGACTGGGGGCACAATTAGAATGGCTGGGTTTAT

1741 TTATTTTTAAAACTATCTTGGTGAGGAATGAAGATGTTTTCAAGATTCCTGGTCTGGGGA

1801 GATGGTCCCTTTTGTCATGCTTTCTGCCTGCTCCCTCTCTGTTGGCTTCAGACATTAGGC

1861 CCTCTCAGAGGCCTGTGGGGTGAGGGTGAGGGATAGCCTGTTTTGGCTGGCTAACAGTAG

1921 ATCTAAGTCGCCTGTGCGTGGGTGCCTTCTTCAACGACTCTTGCAAGCCTGTGTCCCTGC

1981 AGTGGCTTTTCCTCCCCTTGGGGCCCAGTCTACCAAACATGGCCACGCTCACCGATGGTG

2041 TCGCAGGATTTGTGAGCAGAAAGCAGGGCGATGACCGCGCACAGGGACGTGTCTGGGTGG

2101 CTTTGGATGCCTTCCAGCTCGCTGATGGCATCGTGGATGTGGTCTGCAGAAAGAATGTAC

2161 ATAGTCTGATCCTGGGGCTCCAGGCACACGACACAGGGTTCCTGTTGGTCGCTGCCTGCT

2221 TTCACACACGAAATCCAGAACACAGTCTGATGCAAAAGCTCCTCGGGGGGCCTGCATTTC

2281 CTGGAGGGTGAAGGTCTCTGAAGGCCATTGCCACCATGGTCGGGGCCAGCAAGGGGAGGT

2341 CCATGTGTGTACCTGTGCGTGCCGGTCAGAGGACAGACTTAACTGATTTTCTTTAGGCAC

2401 CATCTGCCTTGTTTGATAAATAATGTAAATATTTTATGTGTGTGTAGGAGGCCAGAAGAG

2461 GACGTAAAATCCACAGGAACCGGAGTTATCGATGGTTACGAGAGCTGCCTCGTGGATTCT

2521 GGGGATGGAATCAGAGTCCTCTGTAAGAGCGGCAAGTGCTCTTAACCGTTGATCCATCTC

2581 TCCAGCCCCCACTTTCTATTTTGTTTTTGAGAAAGGTTCTTTTGTATATCGGGCACTTGA

2641 AATTTGATACACAGTCTAAGGTAATGTCGAACTTCCAGAGCCTCCCGCTTCCGCCTCCCA

2701 TGTGCTCCACTACATCTGGCCGACTGGTTGAGAAACTTGAAACCTAGAGATGTGCCTGAG

2761 AGCGAGCCTGGGCACTTTAGAGGGACTGGAGTCAGTTTTGAGGAGGGACCGAGCACTCCC

2821 ACCTGGTTGTTCAAGGCCTACCGATTACATTTATCCCACATGTCCCTAATGTTAGCTACA

2881 GGGTGCTTGGACAGTTCTCAGCACCCAGGCTCTGTTTAAGCCCCACTCCCTCACATCTGA

2941 ACCTTCCAGTGCTTACACCTCCCACTTCTCTTTTCCTCCAGATCCAACTTCCAAGCCCGA

3001 GACGGGTTAAACAGTCCTAGCCTTCTCTCTCTTAACAACCCATTTTATAGATAGGGATAC

3061 TGAGCCTCATGGCAGGGGGCAGTGAGACACGATCTCAAAAGCTAACCTGATATATAGACT

3121 TTACAGGTTTCTGTTCATTAATGGGGGGGGGCTGGGAGGGCCAGGGGTGTTTCCATGGGA

3181 ACATAGCTAGAAGCTGGCATCCAGATGCTGCTAGAAGATAAAAGCTAGGATGTTTCCCCC

3241 TGGGGTAGGAATAATGCCTCACCTTCCCCAAGGATTCCATACGCTCTGAAGAACTGCAGG

3301 GCTGGGTCGCTGCTGTACCTCTCTAGGCCCACATTCGCAGCCTGCAGCACATGGTGGAAG

3361 TACTTTTCCTGGCTGTAGTAAATGATCGAAGCCTGCATGAAAAGAAAGAACATGGTTGCC

3421 ATAGCTCCCGTCCCTCCATCTTTGTTCTGGTCACGATGGGGTGAGACCCACTCCTGTTGA

3481 GGTGACCCATTCCTTCTCACGTCTACAAACAGGAAGGGAGAGGCAGCATTCAGGCCTGGC

3541 CTTCTCCTTCTGCAGCCCTGGGAAGGAAGGCGCTCCAGGGAGTTTGGCTACGGCAGAGGT

3601 TTTAGGTGACTGGGGGATGGTCAATACACCATGTAAGCTTCAAGTCAGGCTCATCCTAGG

3661 CTGGGAAGGAAGGGGACTGAGGAGTGGTCATTTTCAGGGTCTCCATGTCCTCCGCTATAC

3721 ACATCTTGGTATCTTTCACAGCTAAAGAAGCTGAAGCCCAGGGAGACACAGCCCATGGAG

3781 ATCACACAGTAATTGGGGCTGAGATTTCAAAGCAAAAGCTCACTTCCTCTAAGGTTTGTG

3841 GGTGTTGAGTTGGGGATAGATGTGGTTAGGTCGTAAAAGCCTCATGGGCCATTTGAAAGA

3901 GCCTAGACGTCAGACAGGGTCATCAACAGGAACCAGCTCGAATGGCTTTAAGTAGGGGAG

3961 TGAAGTTTTCAGATTTTTAAAACTTTTGGCAGAGCCTGGTGTTCATGTATCCCAGACTGG

4021 CCTCAAACTCAACTATGGTGAGTTTCTGACCCTCCCTCCCCACCTCTCCGGTGCTAGGAT

4081 TAAAGGCACCTACCACCACACCTGTTTTATGGCTATCAGGGCTTGGTGCATATTCTAGGA

4141 ACTGAGCCAGATCCCCAGTTACCTCAAATGATATTCTAAAGAAATGACTCTGGCTGTTGC

4201 GTGCAGTGATGTGTGGGTGGCTGCAGGTGGCACATGGGTAATGCCAGGCAGTAGTGCTGG

4261 TGCAAAGAGCCCAAGTAGAGAAACCAGGCCAGAGGTGTCTAAGGGAGAGATACATGGTGG

4321 CTCTGGAGACTTTGGAAGCTGACACTTCTCGTTAGATTAGGAACAGGGTTGGAGGTGGGC

4381 ATAGACCTCATAGCAGTGGAACACAGATGGCTTAGGTGGGAGGCAGATGCGATAATGGAC

4441 AGGCACGGATTGGTTTGGGGGATAGGATCAGGTCTGGGTGCTAATTTGAGGCTAGGGGTG

4501 CAGAGGACACTGAGGGGAAATGCTTTGGTTAGGGAGCTGAGGAAGATGGAAGGACTTCTA

4561 GAAGCTGCCAGAGTAGTGAACAGAGGGGTAGAGGTGGTTGGGGGGTGGAAGTAGCCTAAG

4621 AGTAGAGATGGCCAGAGGGCAGGCAGCAGGGTGGATGGTGTGGCAGAGGGTGGCCCAAGG

4681 GCAGATGGCCAGAGAAAGCAGAAAGGTAACCCCAGAGCAGAAATGGTCAGAGAGCAGAGA

4741 GCTAGCCCAGGGCAGCAGTCCTGACTGCAGAAGTAGTCCCGAGCGCAGGCGGCCCAGAGT

4801 TCACGCACCATGAGAGAGGAGTCATCGCTGCTCATGGCTGCAGCCCGGCTCAGGTGATGA

4861 CCGGCTGAGCGGGATTCCCAGCTCTGGCCGCCCAATTCGCAGCCCAGCTCACGGCCGGAA

4921 GTCTAAGGCGCGCGCGTCCTTGGCAACCTGGCTGCTGGCCTGTTGCTAAGAACTGTGACA

4981 TCCGGGAGGGCGTGGCCTGGCCCCACGTGACTAGGCCACGCAGCAGCGGGAGAGGGCGGC

5041 C**ATG**GGGCTAGGGGCAAGCAGCGAGCAGCCGGCGGGCGGCGAGGGCTTCCATCTGCACGG

5101 GGTGAGCCGGGGTGGTGGAAGATCACCAGGGAGGGTTGCACAGCCCCGAGGCCTGCAGTG

5161 ACCTCCCTGCATGCCTTGCGAGGCCCAGCGCGTCCAAGATCCGAGGCCCCTTCTCTAAGG

5221 AGCATTGCAAGGGGCTGGGAGAGCTCGTGGGCTTTGGGAAAGCTGCTGAAGAGGGGGTGA

5281 AGTGGCCAGAATGACTGGAGCCAGGGTGGGCATTGGGACCCCAGAGTGAAACATCCCGAG

5341 ACCTAGATGGGGCCCTACGTGTCACTTGCAGGGAGGTACGAGCGAACAGGCTGCTGGGAA

5401 GTATAGTCTGGTTGATAAAGAACGCCGTATGGAGTTGGGGATAGAAAGATCTTTGAACTC

5461 AGCTCCCCTGTTTTGCCCCTTTGAAAAGCAGGTAGATGCAGAGCTGTGTGGAGGCGGGCA

5521 GGTTTGTCAGGTTGTCTGGCTGTTGGGTCAGCCTGCCTGCCTTCATTCCTTCAGACAGGA

5581 TCTTGGTACTTAGTTCAAGCTGTTGTTAAACTCTCAGCTGCTCTTTCTCGGTTCCTCTTA

5641 AATCTTATCTATCTATCTATCTATCTATCTATCTATCTATCTATCTATCTATCTATTTAT

5701 TTTGTGGGTGTCTCATTATGTATCCCCGACTGGCCTGGAACTTGCTATGTAGGCTAGACT

5761 GGGCTGGAACCTACAGAAATCCATCTGCGTGCGCCTGCCTCCTGAGCCATTATGCCTCTC

5821 TGGTCTATGTCAAAGGAAGTCTGGAGTCAGGGCTAGGGAAGCGGTTTCTAGCTGGAGAGC

5881 CACATTGGCTTTACAGATCAAAGTGACAAGATTTGTGCTTGGGGGTTGGCAGACATTGGG

5941 ATGCATCCAACCGGGGGTGTTTTAAAACAGGTTCACGGGAAGTCAGGAGTTTGGGATCTT

6001 TGCACAAGGGCTGAGGGAAGTGAGGTGGTGGGGGTGGTGGACGGAGTGAAGGCAAGGGGG

6061 GCTGGTTAAAGGGGGACAGGTTGATCAGGTCAGAGGATGAGGACCCGTTACAGAGATGTG

6121 TTTTTGGTGTGTGTGTTGTTTTTGTTTGTGCaattgtcgaccatagtgactggatatgtt

6181 gtgttttacagtattatgtagtctgttttttatgcaaaatctaatttaatatattgatat

6241 ttatatcattttacgtttctcgttcaactttattatacaaagttgcggacgcgatcgcga

6301 ccggcggataacaatttcacacaggaacacgagtgtgcaaaccagaggttttagatccct

6361 ttaagttggagttataggcatttgggagctgtcacacttgcatgctggggactcaaccca

6421 ggtcgtctgatagaataataggctcttttaactgctgacctatttctccagtccctttgt

6481 ttgtttgcttgttttttctttgagacagggtttctctatgtagccctggctgtcctggaa

6541 ctcactctgtagatcagactggccttgaactcagaaatctgcctgcctctgcctcccaag

6601 tgctgggattaaaggtgtgtgccaccactgcccggctgtttttgatgtaggatggccttg

6661 agctcttgatacttcccgaatcccaggattacaatgtgcacgagtatacctagctataga

6721 catgtttttgaatatatattctatgccaggcagagcttcaggcactagagataacatggt

6781 agacaaagtactcatatttggaaatcaaacaggggagaaggaaggccataaagagctatg

6841 tggaataggatgtggtggctcatccctggaataccagcattagacgcaaaggcaggagga

6901 ctcccaagtttgaggccagtttaagttatacatcaaggctcttatctcaaaaacatacac

6961 agtgagctgaagagatggctcagcagtcacggacacttgacttctaggaagcccagggtg

7021 acttggtattatgtgcgtgtgcgtgcatgcatgcacaaactgaaggtcatgacattgggt

7081 tgtccacatctgcccgtaagtccagctccaggggacccagggtcctcttctgacttccat

7141 aggcacctccccaccccatacacacagggcatctgtataccctggcacacatgaacacaa

7201 aattttcttttgttttttcaaggtagggttttttttttttttaagccttggctgtcttgg

7261 aactacctcagagatacacttgcctctgcctactgagtgctgggattaaaggcgtgcgac

7321 accactgcccggctacataaatcttaaccacacagcaacggcactgagctccttctccag

7381 gtcctagacttggcattttattttatttgatatttattatatggagctgtggaggatttt

7441 tgttgttgtttttagatttattttcattttatgtttaaggtatgtatgtatgtatgtaca

7501 ctgtgtctggtgtctacagaggtcagataaggggattgcagtcagatggttgcgagccac

7561 cctgtgggtgctgggaactgaacctaggttctctacaagagcagtccagcctctgttggg

7621 acattgtaagtggaggggtgacatgttttacacacacacacacacacacacacacacaca

7681 cacacacacactcatgcatgtgtgaaatggcatgcgtgtgtagaggtcagtggaacttgg

7741 gtttgaatgactctttcacagaggttgcctaagaccattggaatttttttttttttaaac

7801 aaaatctcgattgttcaattttacccatgaaaacttgggagccagatgctggggtgaaac

7861 cctgctagctcagagagacagagaaagtacccagctgaccttctccctcagccttggccc

7921 cagcagcaatgccctctcacactgtctcaaaaaacctctttcaaaccgaatatcctttcc

7981 tacttcgtatgtatctctctgtccaacctcctgaccccatcttagtctaaataatcctat

8041 gcccaggtcatgtcaactggttgcttgttctacctcttaacctttggttggctgtattta

8101 atcctgtttataatattcaagcagaaagctctcggattaaaggtgtgtgccaaggctgag

8161 tcacacaacaactaaaagcagattttccggtaaattaacacagtctcggggtccacagtg

8221 tgttaagtgtcctgtaacaggaaaactcagacatttacattatgattcagcacagtagca

8281 agattagttatgaagtagtaatacaattttatggggtcagcacaacacaaggaactgtat

8341 aaaaggttaacagcattgggaaggttgagaactaaaggaaccctgaagcagggacccctg

8401 agggatggagcaggactaggagtccccaaacagtccaggaatgcttgcccttggacttgg

8461 ccaagcccccaccataggcccactcccactagaggccgtggcagctctagcaagtgccac

8521 tcagggactcagttgctgttgctgtccgggagtttttatgccggggcaaactggccttga

8581 actaaaggcacctgtaacacttggttctcttcaggctcctgaagagttctgcagcttggg

8641 gaatctagtgtaggtatctaggatctgaggtcacctgagtcctgtgccctgtcatcggca

8701 ctgttaccttggggcctggttgatgcctggcccaagaagttcatagcaaaaggcttgagt

8761 caaaggcctaggtgcatctctggagttcaggacctggggtaaaggttgccttgtttgagc

8821 aaggccctgtaggtggggcctggcgatgtgttcctggtacagcttgtgatgggggagctg

8881 gctgtactttggagacagcagtaagttagtaattgagaggggcagggagcaccgagagca

8941 ggggtggggagcaccgagagcaggggtgggtgcagctgagcgcatgtcctgaccctggga

9001 tccgctctccttcactgtcagcctgtccctgcctgctcaactggagctaggcagctactc

9061 agggtctaggcgcttcagaatctggccacaggctgccttgcccagcccttatagcaaggt

9121 ctggctgcatttctcctgcgtgtctgggctttcctgcccaatcccagttttggggtgggg

9181 gtggggtgatatcctttaggtgccccttactccagggctatctgagtcctttctggaagc

9241 attcgcgtatcacggttgggaaacttatgtcagggaaggaggtgagtgggggggtgggtg

9301 agcagagcagtctgcggaggtgagaccttcccactggtccagggtgggggctgggctcct

9361 tgggccgtgacgcacctctgctctggaacaggtacaagagaactcgccggcccagcaggc

9421 aggcctggagccctacttcgacttcatcatcaccatcgggcactcgaggctggtgaggct

9481 ccccactgccccgtccccacaggctgctcctgttcgcttctgcctccttttttcttcccc

9541 tgtgaccagggccacagagggacatttggaggcatccagcaggcctaagaaggctgcctt

9601 tcaagacatatgaggccttaggatgtggccctaagaggccttttgcttgctttgttagtt

9661 ttctggaaggcttggggaccaaagcttggggtgcctgaactgggtgatgctggctcaggt

9721 gctgcctggagctgggtggtggctgtggtggctgcaggcccaaacctagcctgtgaagcc

9781 gggcccagcaatcgtttcagtttgccctcacaccctgtgctccatgcctctcagtccctg

9841 accagtctaagggttctggcccaggggtgggaggtctcagacagagccagctcctccctg

9901 accctgcagaacaaggagaacgacacgctgaaggcattgctgaaggccaatgtggagaag

9961 ccggtgaagctggaggtattcaacatgaagaccatgaaggtgcgcgaggtagaggtggtg

10021 cccagcaacatgtggggcggccagggcctcctgggagccagcgtgcgcttctgtagcttc

10081 cgcagggccagcgaacacgtgtggcatgtgctggtgagtcctggtagggtgtgtggagtg

10141 ggcggggtctggcacacaggagcaggatggccagggctgatgggtggggcagggatcaga

10201 gggcagtgctgtgggatctagttttgttccagtgtcctcagaggacagcgggtggatcgg

10261 tgcttttgttaaccgtgtgaccttgggcatacttgtttcatgttactgttttgggacagg

10321 gttggaattcactatgtgtagccagattggccataaacctgcagagacctctagcctctg

10381 tctcctgagtgcagggactaaaactgtgtgctagcacacagtccacaatctaaccctagg

10441 ctgcactgcttgctccactggcctcagaagaagcaagtggatcttactgagttcaaggcc

10501 ggccagacctgcatagagagttccaggttggccaaagctacatagtgagaccctatttca

10561 aaaaaatttaaagaaaaccatctccctgccaagagagtttatggcagagtgaagtgtaag

10621 cgttgaggtcctgggtctggatccccagcagctatgaaaaagccaggacagcagcattca

10681 gcaaggacactgtgtagagtgacagaggacacctggccaccacacttactcactcaaaat

10741 tggggggtggagggtaccaaggtaaaggtccttgccaacaggcctggcaacctgcgttca

10801 cttgccttgctgcctgagcccaggcacgcctctgatagactggaagctaagcacaaggct

10861 ccctccctggaaggctgctggagcctgggtgtcctacacatggcctgtggccacgagcgg

10921 caaggacagtcctaacacagtccaacccaaagccataaactgctaaaaccattacgaggg

10981 gccttgcggagtgttcacctgtactggagttcaagctccggaagccaggtggtgtgcact

11041 tgtgactggctagggacaggcgagatgggcagctcccaggaactcactgccaggcatgga

11101 gtctaattgctaagctccggccaaagagagaatgcctcaaaggaggtgaacaagattccc

11161 aatgatgacacccaaggatatcttggcctccacaggcacacacagaatgagcacatgtac

11221 acaggcacacacacacacacacacacacacacacacacacacacacacacagcgagaaaa

11281 agagcacgtgtccacaggcacacactttttttttaatgattggctatggagatagcttaa

11341 tgcatgaataatgtcaatcaaacctgaattcagtccccaggaacccacactgtggaagga

11401 ctgttgtcctctgacctccacgtatgccagggcacacgtgcacccacacaagtaagtgtg

11461 gtggtgtttgtaaacaaaagcaaagcactaattctgaggtgtggcttgtaaggaggctgt

11521 gtggttctgtagcatgagcttcgagatggcagcatcctgcctgctacaccatcacaccaa

11581 gtaaagagaggcaggctcttggaacccacaagagcatatgacccaaaacaagtagccacc

11641 ccaagccagaagctggacagccctgccacgaccacactgctcttgagggctccggagtcg

11701 gccttgtcagccctttgtgcacgggcacgcctttgcacgcagaccgcaaagattctgcag

11761 aggcagctagaacccaaccagcaagccttcccagcaggggcaccttccctccatagaaca

11821 ggagactgaccattgcagggctctggtgtaccaccgatacacacagtgtcaggtgggcat

11881 atgagtctgagagctaaggctaacatgtggggcagctcagccactgagattcattcctgt

11941 acccgttatatagcaacaggccagagaaactactatgccatctttataaggcgcataacg

12001 ataccacgatatcaacaagtttgtacaaaaaagcaggctggcgccggaaccgaagttcct

12061 attccgaagttcctattctctagaaagtataggaacttcgaaccctttcccacaccaccc

12121 tccacacttgccccaaacactgccaactatgtaggaggaaggggttgggactaacagaag

12181 aacccgttgtggggaagctgttgggagggtcactttatgttcttgcccaaggtcagttgg

12241 gtggcctgcttctgatgaggtggtcccaaggtctggggtagaaggtgagagggacaggcc

12301 accaaggtcagccccccccccctatcccataggagccaggtccctctcctggacaggaag

12361 actgaaggggagatgccagagactcagtgaagcctggggtaccctattggagtccttcaa

12421 ggaaacaaacttggcctcaccaggcctcagccttggctcctcctgggaactctactgccc

12481 ttgggatccccttgtagttgtgggttacataggaagggggacgggattccccttgactgg

12541 ctagcctactcttttcttcagtcttctccatctcctctcacctgtctctcgaccctttcc

12601 ctaggatagacttggaaaaagataaggggagaaaacaaatgcaaacgaggccagaaagat

12661 tttggctgggcattccttccgctagcttttattgggatcccctagtttgtgataggcctt

12721 ttagctacatctgccaatccatctcattttcacacacacacacaccactttccttctggt

12781 cagtgggcacatgtccagcctcaagtttatatcaccacccccaatgcccaacacttgtat

12841 ggccttgggcgggtcatccccccccccacccccagtatctgcaacctcaagctagcttgg

12901 gtgcgttggttgtggataagtagctagactccagcaaccagtaacctctgccctttctcc

12961 tccatgacaaccaggtcccaggtcccgaaaaccaaagaagaagaaccctaacaaagagga

13021 caagcggcctcgcacagccttcactgctgagcagctccagaggctcaaggctgagtttca

13081 gaccaacaggtacctgacagagcagcggcgccagagtctggcacaggagctcggtacccg

13141 gaagatctggactctagagaattccgcccctctccctcccccccccctaacgttactggc

13201 cgaagccgcttggaataaggccggtgtgcgtttgtctatatgttattttccaccatattg

13261 ccgtcttttggcaatgtgagggcccggaaacctggccctgtcttcttgacgagcattcct

13321 aggggtctttcccctctcgccaaaggaatgcaaggtctgttgaatgtcgtgaaggaagca

13381 gttcctctggaagcttcttgaagacaaacaacgtctgtagcgaccctttgcaggcagcgg

13441 aaccccccacctggcgacaggtgcctctgcggccaaaagccacgtgtataagatacacct

13501 gcaaaggcggcacaaccccagtgccacgttgtgagttggatagttgtggaaagagtcaaa

13561 tggctctcctcaagcgtattcaacaaggggctgaaggatgcccagaaggtaccccattgt

13621 atgggatctgatctggggcctcggtgcacatgctttacatgtgtttagtcgaggttaaaa

13681 aacgtctaggccccccgaaccacggggacgtggttttcctttgaaaaacacgatgataag

13741 cttgccacaaccatggaagatcccgtcgttttacaacgtcgtgactgggaaaaccctggc

13801 gttacccaacttaatcgccttgcagcacatccccctttcgccagctggcgtaatagcgaa

13861 gaggcccgcaccgatcgcccttcccaacagttgcgcagcctgaatggcgaatggcgcttt

13921 gcctggtttccggcaccagaagcggtgccggaaagctggctggagtgcgatcttcctgag

13981 gccgatactgtcgtcgtcccctcaaactggcagatgcacggttacgatgcgcccatctac

14041 accaacgtgacctatcccattacggtcaatccgccgtttgttcccacggagaatccgacg

14101 ggttgttactcgctcacatttaatgttgatgaaagctggctacaggaaggccagacgcga

14161 attatttttgatggcgttaactcggcgtttcatctgtggtgcaacgggcgctgggtcggt

14221 tacggccaggacagtcgtttgccgtctgaatttgacctgagcgcatttttacgcgccgga

14281 gaaaaccgcctcgcggtgatggtgctgcgctggagtgacggcagttatctggaagatcag

14341 gatatgtggcggatgagcggcattttccgtgacgtctcgttgctgcataaaccgactaca

14401 caaatcagcgatttccatgttgccactcgctttaatgatgatttcagccgcgctgtactg

14461 gaggctgaagttcagatgtgcggcgagttgcgtgactacctacgggtaacagtttcttta

14521 tggcagggtgaaacgcaggtcgccagcggcaccgcgcctttcggcggtgaaattatcgat

14581 gagcgtggtggttatgccgatcgcgtcacactacgtctgaacgtcgaaaacccgaaactg

14641 tggagcgccgaaatcccgaatctctatcgtgcggtggttgaactgcacaccgccgacggc

14701 acgctgattgaagcagaagcctgcgatgtcggtttccgcgaggtgcggattgaaaatggt

14761 ctgctgctgctgaacggcaagccgttgctgattcgaggcgttaaccgtcacgagcatcat

14821 cctctgcatggtcaggtcatggatgagcagacgatggtgcaggatatcctgctgatgaag

14881 cagaacaactttaacgccgtgcgctgttcgcattatccgaaccatccgctgtggtacacg

14941 ctgtgcgaccgctacggcctgtatgtggtggatgaagccaatattgaaacccacggcatg

15001 gtgccaatgaatcgtctgaccgatgatccgcgctggctaccggcgatgagcgaacgcgta

15061 acgcgaatggtgcagcgcgatcgtaatcacccgagtgtgatcatctggtcgctggggaat

15121 gaatcaggccacggcgctaatcacgacgcgctgtatcgctggatcaaatctgtcgatcct

15181 tcccgcccggtgcagtatgaaggcggcggagccgacaccacggccaccgatattatttgc

15241 ccgatgtacgcgcgcgtggatgaagaccagcccttcccggctgtgccgaaatggtccatc

15301 aaaaaatggctttcgctacctggagagacgcgcccgctgatcctttgcgaatacgcccac

15361 gcgatgggtaacagtcttggcggtttcgctaaatactggcaggcgtttcgtcagtatccc

15421 cgtttacagggcggcttcgtctgggactgggtggatcagtcgctgattaaatatgatgaa

15481 aacggcaacccgtggtcggcttacggcggtgattttggcgatacgccgaacgatcgccag

15541 ttctgtatgaacggtctggtctttgccgaccgcacgccgcatccagcgctgacggaagca

15601 aaacaccagcagcagtttttccagttccgtttatccgggcaaaccatcgaagtgaccagc

15661 gaatacctgttccgtcatagcgataacgagctcctgcactggatggtggcgctggatggt

15721 aagccgctggcaagcggtgaagtgcctctggatgtcgctccacaaggtaaacagttgatt

15781 gaactgcctgaactaccgcagccggagagcgccgggcaactctggctcacagtacgcgta

15841 gtgcaaccgaacgcgaccgcatggtcagaagccgggcacatcagcgcctggcagcagtgg

15901 cgtctggcggaaaacctcagtgtgacgctccccgccgcgtcccacgccatcccgcatctg

15961 accaccagcgaaatggatttttgcatcgagctgggtaataagcgttggcaatttaaccgc

16021 cagtcaggctttctttcacagatgtggattggcgataaaaaacaactgctgacgccgctg

16081 cgcgatcagttcacccgtgcaccgctggataacgacattggcgtaagtgaagcgacccgc

16141 attgaccctaacgcctgggtcgaacgctggaaggcggcgggccattaccaggccgaagca

16201 gcgttgttgcagtgcacggcagatacacttgctgatgcggtgctgattacgaccgctcac

16261 gcgtggcagcatcaggggaaaaccttatttatcagccggaaaacctaccggattgatggt

16321 agtggtcaaatggcgattaccgttgatgttgaagtggcgagcgatacaccgcatccggcg

16381 cggattggcctgaactgccagctggcgcaggtagcagagcgggtaaactggctcggatta

16441 gggccgcaagaaaactatcccgaccgccttactgccgcctgttttgaccgctgggatctg

16501 ccattgtcagacatgtataccccgtacgtcttcccgagcgaaaacggtctgcgctgcggg

16561 acgcgcgaattgaattatggcccacaccagtggcgcggcgacttccagttcaacatcagc

16621 cgctacagtcaacagcaactgatggaaaccagccatcgccatctgctgcacgcggaagaa

16681 ggcacatggctgaatatcgacggtttccatatggggattggtggcgacgactcctggagc

16741 ccgtcagtatcggcggaattccagctgagcgccggtcgctaccattaccagttggtctgg

16801 tgtcaaaaataataataaccgggcaggggggatctaagctctagataagtaatgatcata

16861 atcagccatatcacatctgtagaggttttacttgctttaaaaaacctcccacacctcccc

16921 ctgaacctgaaacataaaatgaatgcaattgttgttgttaacttgtttattgcagcttat

16981 aatggttacaaataaagcaatagcatcacaaatttcacaaataaagcatttttttcactg

17041 cattctagttgtggtttgtccaaactcatcaatgtatcttatcatgtctggatccggaat

17101 aacttcgtatagcatacattatacgaagttatgtttaaacggcgcgccccggaattcgcc

17161 ttctgcaggagcgtacagaacccagggccctggcacccgtgcagaccctggcccacccca

17221 cctgggcgctcagtgcccaagagatgtccacacctaggatgtcccgcggtgggtgggggg

17281 cccgagagacgggcaggccgggggcaggcctggccatgcggggccgaaccgggcactgcc

17341 cagcgtgggcgcgggggccacggcgcgcgcccccagcccccgggcccagcaccccaaggc

17401 ggccaacgccaaaactctccctcctcctcttcctcaatctcgctctcgctcttttttttt

17461 ttcgcaaaaggaggggagagggggtaaaaaaatgctgcactgtgcggcgaagccggtgag

17521 tgagcggcgcggggccaatcagcgtgcgccgttccgaaagttgccttttatggctcgagc

17581 ggccgcggcggcgccctataaaacccagcggcgcgacgcgccaccaccgccgagaccgcg

17641 tccgccccgcgagcacagagcctcgcctttgccgatcctctagagtcgagatccgccgcc

17701 accatgattgaacaagatggattgcacgcaggttctccggccgcttgggtggagaggcta

17761 ttcggctatgactgggcacaacagacaatcggctgctctgatgccgccgtgttccggctg

17821 tcagcgcaggggcgcccggttctttttgtcaagaccgacctgtccggtgccctgaatgaa

17881 ctgcaggacgaggcagcgcggctatcgtggctggccacgacgggcgttccttgcgcagct

17941 gtgctcgacgttgtcactgaagcgggaagggactggctgctattgggcgaagtgccgggg

18001 caggatctcctgtcatctcaccttgctcctgccgagaaagtatccatcatggctgatgca

18061 atgcggcggctgcatacgcttgatccggctacctgcccattcgaccaccaagcgaaacat

18121 cgcatcgagcgagcacgtactcggatggaagccggtcttgtcgatcaggatgatctggac

18181 gaagagcatcaggggctcgcgccagccgaactgttcgccaggctcaaggcgcgcatgccc

18241 gacggcgaggatctcgtcgtgacccatggcgatgcctgcttgccgaatatcatggtggaa

18301 aatggccgcttttctggattcatcgactgtggccggctgggtgtggcggaccgctatcag

18361 gacatagcgttggctacccgtgatattgctgaagagcttggcggcgaatgggctgaccgc

18421 ttcctcgtgctttacggtatcgccgctcccgattcgcagcgcatcgccttctatcgcctt

18481 cttgacgagttcttctgagcgggactctggggttcgaaatgaccgaccaagcgacgccca

18541 acctgccatcacgagatttcgattccaccgccgccttctatgaaaggttgggcttcggaa

18601 tcgttttccgggacgccggctggatgatcctccagcgcggggatctcatgctggagttct

18661 tcgcccaccccccggatctaagctctagataagtaatgatcataatcagccatatcacat

18721 ctgtagaggttttacttgctttaaaaaacctcccacacctccccctgaacctgaaacata

18781 aaatgaatgcaattgttgttgttaacttgtttattgcagcttataatggttacaaataaa

18841 gcaatagcatcacaaatttcacaaataaagcatttttttcactgcattctagttgtggtt

18901 tgtccaaactcatcaatgtatcttatcatgtctggatccgggggtaccgcgtcgagaagt

18961 tcctattccgaagttcctattctctagaaagtataggaacttcgtcgagataacttcgta

19021 tagcatacattatacgaagttatgtcgagatatctagacccagctttcttgtacaaagtg

19081 gttgatatctctatagtcgcagtaggcgggttcatgctggcccacccttcaaataaggaa

19141 caaagccacgggggctgcacaggcagctcacaaccatctgcaactccagtcccaggggac

19201 ccaaggtcctcttgtggcctccaggagcactgcacacatgtggtgcacagataaacatgc

19261 agataaaatgcccatatatacaaagtaataaaatttaaaagaaactaaggtccagagtta

19321 agaatgcccaagagtgctgccatctgttcaccaggcagtaacttgggggaggggagaaac

19381 ttgggccctgtggagaagtctttcttgaggccctgctgggcaatagagggcaagtgtccc

19441 ctccctagagaagcctcttctctctgcttagcgttgaatcttagtttatcattctcactt

19501 caggatgtggagccctcttcacctgctgccttggccggcctgtgcccttacacagactac

19561 atagttggctctgaccagattctccaggaggtaaagaacccatggcgtgcttgtttccag

19621 gtcccctgaagggctgtgggcagacgcgttgcacttgtgtctcaatgctgggaacacccc

19681 tccaacagacactgtctttctcctagtcagaagacttctttactctcattgagtcccatg

19741 aggggaagcctctgaagctgatggtgtataactcggagtccgactcctgccgggaggtga

19801 ctgtgactcccaatgcagcctggggtggagagggcaggtacttcctggggtcagagggct

19861 acagggcgaggtgggtgggctgtcctagtgggcatgggctcctggcatcccttggaggaa

19921 acagcccggaccgctcatccttgcccaggatccttgctcagtcactgttgctggttgggg

19981 gagggttatataggtagggagggctgaagtgtgggctaccctgatggaacacatctggct

20041 ttccacagtctggggtgtggtattggttatgggtacctgcatcggatcccaacgcaaccc

20101 tccagccagcacaagaagccacccggtgccacaccacctggcactccagctacgacctca

20161 caacttactgcctttcctcttggtgccccaccaccttggcctatccctcaggactcttct

20221 ggcccagagttgggttccaggcagagtgacttcatggaggtatgtgggagcacctgtcag

20281 gccagggaagagaggagtgtctggggcattgggcctgccacctagaggctatctagggac

20341 cgtagaaacactgtttgtgggcctgtgagttccttgatgttagggggcccatctgcttct

20401 agcatatagaagtagagaggctctgcttgagtaaaacatgcttttggctctattgtcctc

20461 gagtggaggtgaggcacaggtagtcatttatgagttggaaactgtgactcagcgatgggg

20521 agaaccttgtgccgcagtgaggtgggggcagccaggccagtgctagacatggtaggccgc

20581 atggtccccatttgcacctggattcaagggaggttcagagaacaggggcattacctgtgg

20641 gtacctggggatcccctcggggagcggcagaaagcttccccagtatgtgtttgtagggag

20701 gatctggctggcctcccatcacggggaggaagggcagtgcttgccgtgcaagggctctag

20761 ttaggttgaagtgaacatctgtgcaccggaggtttctaggcactggcctaactaaccctc

20821 agtttctcccttcctggtacctctaggccctaccacaagtccctggtagctttatggagg

20881 gacagctccttgggcctgggagtcccagccatggcgctgctgactgtgggggatgcctgc

20941 gtgctatggagatcccgcttcagcctccacctccagtgcagcgggtcatggacccaggta

21001 tggggcaggtcctgaaagagactgcagtacctagagccctgatgctaaagtgggctacag

21061 gctcacagctggacttgcacagcagtgccccagctttcctcaggacctgctagagatggc

21121 gcaacgcaattaatgataacttcgtatagcatacattatacgaagttatggtctgagctc

21181 gccatcagttcaagtccagagtagaggaggcccactctgggtgacttggtagatagaggt

21241 aaagcaggatttgggccaactgccatggtattactccatgatgttcagctctgggttctt

21301 tctgtcccttagacacaggtggtgggagtctcgatggggttggagggaaggcatctgtgg

21361 ggctgagggcgagctaggtagcccaggaatatggcagagggaaggcaggctcccatatct

21421 ccagtagttggaggttttttgttttgtttttttttttttttgcgagcaaagaactgggtt

21481 tgctttccctctccaggcttcctggatgtatcaggcatgtccctcctggacagcagcaac

21541 ataagcgtgtgccccagcctgtcatcttccacagtgctgacctccacagctgtttcagtc

21601 tcaggaccagaggacattggttctagcagcagttctcacgagcggggtggtgagtggcct

21661 ttagtgatgccgtctgagaaatcctgtgtttggggagggctgccccgagttccctaggtg

21721 ggctagaggttggctgtatcaagggattcacatgagtgacttcctctgactccaccaaag

21781 cctgggctgcaggggtcatcaccgagggtgggtctcctcagccccacttcatgtttcagg

21841 tgaagccacgtggtcagggtcagagtttgagatctccttcccagacagtccaggtgccca

21901 ggcccaggcggaccacctgccccggctgactctccccgatggcctcacatctgcggcctc

21961 acctgaagaagggctgtctgcagagctgctggaagcacagactgaggagccggcagacac

22021 agccagcctggattgcagggcagagactgaggggagagccagccaagcgcaggccacccc

22081 agatccagagcctgggctg**tga**gaacacccctggtgacatttggtgtggcttagctgggt

22141 agctctctaggctgcaggctcttgtgaggcacgggcattgctacaatacatacatggtcc

22201 tgggtcgaagggacagcacacccgagcagcaaccagcttctgtagaggcaggtgacggaa

22261 tctagactgtcaatgaagcatcgatgccccatcacaatatccaggtgaagatggggcgag

22321 ggacaagaggctgtgtccataggcagccacacctgtactgaggaagaccctaagtcccaa

22381 tgcccttcctcactctaatccttacactggatgagcgaggtggtatatctggccacttta

22441 tttcttaggatgggaaattctagggatttaccttcagagggatctacctccgctagctgt

22501 tcacagccccagggggaggtacaaaggagcttacactccagttcttgggtcctatgacgc

22561 tcccttcagccctcacgtagacagctggctctggtggcagacgggtcaaactacgcttcc

22621 tgtcaaaacccctccacctgcccgtgctcagggttcttctcctgaagagctggaaaaggc

22681 ccctcccacctgtcctcagcgtaagggaaaggagcctttacaggctcagtctccgagagg

22741 acagctgcaccgctgccttgctacagatggtgccaggcatgtcctctgcagccccagttc

22801 catgtcctttgccaatcactgtaagctgcctttggtcttcttttccagcctctttgttcc

22861 atcatccacagaccatgtaactgtctgttcagggtacttaggctggtaagccaaggactc

22921 tgttccaagaagcacaggccaaatggttcagtgggccaggtcctggatgggcaaacagaa

22981 cccaaagcactaggaatacatttatagccaggcctgtgaggcccccgctccccaggggcc

23041 actgagaggcctgaggccgtacaacatggcttaccacagggctgctctggggccagctca

23101 atttagctgtggttaggcactctgacccccagctgccttcattggaaggttctgctcccc

23161 aggaccccatgtggggtgaaacggaggctgatgctgtttcaagtatgaacccccagatgg

23221 tttctttccctgtaatctgctatggggaaagtgacaagtgaaaaaataaacatgtccagc

23281 acactgaagtgtttgtaactaaagctttattctatacaacccgagatacagactctgccc

23341 ctttggcgttgcagactgtccagttttctggaaacttagtactgatttttgactagctgg

23401 ggaaaaaattacaaccatgctggggtaaactcctccataggggcacaactgcctgtggat

23461 cagtactgcgccagtgaaccaaccagcaaaccctacgcttagtgcagaggcccctcctgc

23521 aaacacgaccaccccaccccctgcttttactttatagctatcactaaaaaatatgcacca

23581 aaagtgcaccgatgtgtccagcatagaaaggacttctgtagaaaagctggctactctagg

23641 gcggagaatggtttaacactggcaagtgttcttgctcacttgagggtatggaagccatgg

23701 cctgtctgtgttccagagttaagtgaggggaccctgttctgcagtcaatttgtaagggac

23761 agatctgggcccctcagctgttccctgcaggacctgtgagatagagagaaaacatctttc

23821 ttccccaagcccccctcccctgtgctcctgtgggcagggaccacacacacaaggcaggag

23881 cagacggacggatggggaaagctgcaccccccacccccaaggcacgtgtgcttccgctgt

23941 gctcaaaggttcatctcctgcagcttcacgggctggaccatttgtctgaatgtaaatgac

24001 cttgggaagaatccaaagtagtttttgccaaaagttgaagtgtttatccccaataagtta

24061 atattctgtacaagatccaagtgagtctatgaaagcttttgactgttagaaagacggcag

24121 aaagacctggacgaaggcgtaacaaaagcccgtgtataaaactggaaaaacaaacatgaa

24181 ccacaatataaatatcaaaccaaCTCACTGTGAGATCTTTTTACTCTAGTAACATAAACT

24241 TTAGGTAGCATCTGAATGAAACTGCAACTTCAGCATGGCCGTAAGTTCTGACAGTTGTCG

24301 TGCTGCTCACGCGGAGCAGGAAGCTGTGGCTTCGCTGGCTTTGCCCGCGAGGAAGACTGG

24361 CGGAAGGAGTGGCCACAGCAGCTTGGGCTATCGGACAGCCAGGGCGCCATGGGCAGCACT

24421 GCGCAGCACAGGCTGTGGGTGGCAGCCTGCAGGTCCACGTGCTGCGCAGGCTCCAGCCCT

24481 GGAGTTGCAGGTCTTCCTACAAGAGCACAGCTGAGACGTGGCATTTCAAAAGTAGCCCAA

24541 CATGGTCATTTCTCAGGAAATGGGCTGGCTCTTGAATTGTGCAACAGTGATTTCCATTAA

24601 AAATAACTCTTACATAGTGAAAACAACTTGTTTTTTTTTATATAATGACAAATTTGGAAA

24661 AAGCCTTCATAGGTACTGCATGAGTGGAATGTACTTGGAAAAGCTGCAGAGCCTGTGGGT

24721 TTGAAACTGAAACATGGCGACTGATGTGGCCCCGCTGATGTTCTCTAACAAGTGCTAAGA

24781 GACAAAGGGGGGTCCTTCAAAAGGAGTCAGCGAAGTCTGCACTGGCCTGTTGTAGATTCT

24841 CTGTACATCTCCCCGGACAGTGCCAGGGGCTTGTCCTTGCACTCTTGAGTCCCTGGGGAC

24901 ACCTTAATCTATGCATGGAGCCTGGTTTCTATATTACATCTCCATTAGTCACGTCACCAA

24961 ATAGGCCGGTTGGAGGTCTTGCTCCCGTAGGACGTGGTGGGCTTCCTAGGACTACTCTTG

25021 GGGCCACAGAGAAAGGTGGTTGGTGAAGAACCCAGGAGCATGGGCCCACCCTCACGTGGA

25081 CTTCTGACGGTAATGGAGGGTGAGGTCCCCACCACTCTTCCATATGAAGTGCTTTACTGT

25141 CCGAAGGTCCATATTTGGGTCCAAAACCTGAATAGCCAAAGTGCATTGAGAATGTCAACA

25201 TCACTAACAGCTAAAGTTAACCAGGGACTCTGCCAGGTGGTCCAGCTTGCTCCCCACTGT

25261 GTCTAGGAGCCTGGTTTTTGCTTCCATACATGGAATCTGAAATGTGTACCTCTGCGGCTC

25321 ACCAGCTCAACCCTGCCTGATGACAGAGTGCTTGTCTTGGTTGTAATTCATCGCTTCTCC

25381 ACCCACCTCTACTCCAAGTTCTTTACTGTGACAGATAGCACCAGTTTACCCAGCACAGCC

25441 CTACTATGAGCATCTGGAAATCTGTCCTTTCCCAAGTACCTCCCTCAGGAGAGGGAGCTT

25501 CAGGTGTGCTGAAGAAAGTTTCCAAGAGACACAGCTCTCCAGACACCTGAGAGGCCACAA

25561 AGCACGTGGTCTGTGACTTTATGGGCCCCTCTGCATGGGTCCACTTACCTGGTCCTGGCA

25621 CAGAAGTTCAATTTTCTCCTCTGCCAATACCGCGATGTCCTCCTCCTTCTCCTGCTCTGG

25681 CTTTTCATTATTAGAAGAGCTGGTGGTTTGGGACTCATTGTCCAGGTTGATAATCTTCTC

25741 GTAGACGTGCTCCATCACCTTCCGGACCTGCAGCATGTCACTGGCAGACAGCCTGTCTCT

25801 AGAAAAGATGGGTTTTCATATCAAAAATCTCTTATTATTTTTCATTTGTGTCAATATCTC

25861 AAGATAAATATAAACAGACATCTGAAATCAATACATGTTTTCAGTCATTGCTCCGAATCA

25921 CTTGAAATCTGTCAGGAGAGATCCTGTGGTGCCTTCGAGGGCTCATGCACACACAACAGC

25981 CACTTGTACTATATGCCTCCAAGCCCTCGGCTAAAGCCTGGGTAGACCCACCAGTTAAGG

26041 ACCAAGGGCTCAGTGTTATCTCCTACTGCTGTGCCTTCAACTAGGAGACAATGAATACCC

26101 CAGGCTAGACATTAAGCCACAGCTTGCTCAGACCACAAACCCAGCATTACACAACTGAAG

26161 TCCCTCACTTTTAAGCCCTTATCTCTGATATCAGTAAACTACTATCCTATCTCACAGCTA

26221 CAGGTCAAACTGTTCAAGGATCTTCAAGTGGAGTCATTAAATCCACTTGAAGTGGCGTCT

26281 ACGGCTTGCTGAAGTGATCCAATGCGTGCATGTGGAGGAGGTGGAGGGGCAGGCGGAAGT

26341 GTGGGTTGGACACTATGTTTCAGTATTACAGAGGTTTGTCCTCATTTGCTGGGTCACCAG

26401 GAACTTGTCCTCTGTGTGAGCTTGGGAATCCCTCTGACAGTTTTAACAGCCTCGTTGATT

26461 TCACTCGCTAGCCATACACACCGATTATGGTTCAGACTCCAAGTCCTCTGCTGTAAGCTT

26521 GTCTTTGAGCCATATCTACCTAGCAGGAATTAGTGGAACTGCACTCTGTGACCTGGCATT

26581 GAAATTCCGAGTCAGGAAGCCTCTGACTCAGCTGTATCGTTTCACGCCATCCTATGTATG

26641 GGAGCACACATCTCTATACCATTTCTGGTACCCGAGCAAGGTCCTGTTTGGGAAGTCTGT

26701 CTTTGGGTACTCCACCCATCCATGTGTGTTCAGGTGAGATTTCTTGTCCTCCTAGAAGGC

26761 TCTGTTCAGAGCTGGTTTGCTCCCACTTTCATCCTTTCCTAAGGTCTGTGAAAGCAGCCA

26821 CTACACACAGGTTTTTATCCTCAAAGGAACTGTTTGGTTTGGAGACTGTGGATCCCCTCT

26881 GTTTACCTCCTAAGAACTGGGATTATAGGTGTGTGCCATCACACATGGCTACATGGTCTT

26941 GCTATGTAGTCTTGGCTGGCCTGGAACTCACCATTCTACTGCCCAGGCTGGCCCCTGCCT

27001 CTGCCTCCAAGTGCTAAGATTTAAAAGGCAGTGTCAGCAACTATGGGTTAAACTTAATTT

27061 TGAGAGGAGACAAAACCCAAAGCAATGGCCTTCAACCTAGCACCTGCCTCCATCTGATAA

27121 AGGGAGCTAAAGGACAGAGGCACCGCAGTGACCAGCGCACGGAAGCACAGCAGTTACTTA

27181 CTTTTTTAAAGTTTTTGCTCCTGAAGATGCATGAGGTTGGAGGTAGAAAGGAATTTTGTT

27241 GAATTTGGGCATATTTTTCTGAAATATTAAAAGTTTAAGAGGTATAATAGTTTCTGTTAG

27301 CTGATAAAACTAAAATGTTATCAAAAGGAAAACAATTTACAAATAACCATTTTTACAATA

27361 AAAGTGACTTTTAGTGAAATTTATAGTAAGTTATCAATAGAAGTATGCCTTAAGAGAAAA

27421 TTTCTCAGCAAGTATGCTTAGTTTACAATAAAAATAGATGAAGACAAATTATATATAGTA

27481 GTCAACAACAGTGGCACAGCTCTTCTTAAGTTATCATTTAAGGCAGCACAGGAAAACAAA

27541 CCTACATTTTTAAAGTTCCATAAATCAAAATATCTGATGATTGCAGTGTTTTGAGAACAC

27601 CCCAGAACGTTCACCTTTGAGCAGACACTTGGGCACTGATGGCCCATCGTCCACTGGAGA

27661 GGCTATCAGGCCCTCCTACCATCTACCTCTTCCTCAGACAGAGGGAGGGCTACCGTGACA

27721 GCTGAAATGATCCAGCCAGTTTTGGAACACAGCTCATGTCATAGGGTCACTACTGCACCC

27781 AAGTGAGTTCTATACTCAAAAGAAACAGTGGTTTATGGGAGCCTGGACAGGGATGGCCAC

27841 GGACACCCAACAGGTGTCACTCTAGTTCTAAGTTCTTGAGGTGTATTTGGGATAGAAAAG

27901 AATTTTTTTTTAAGTTTCAAGAGATTAAATCCCTTTTCATATGGCTTTAGGGATATACAA

27961 GTAGTCAAGAGGACACAGAAAGTCTAGAAACAAACTGAGTCTGCAGGAATTTAGTACACT

28021 ATACAGACAGCATAGCATTTCAAATTAGTAGACCGGACAGATTCCTATAAACTGCATTGG

28081 CATATGGCTTAGTTCACTGGAAAAAGAATGTCGCCTTACAGCAAACAGAGAGGCAAGTAA

28141 GAAAACAGTCAAGTCAGAGTTGATATAAAGGATATTCTAAACCGGGTGATCTCTAACAAA

28201 ACATGGACCTCAGCGCCATAAAACCCACTTTGACTATATAACAAGTTCCCACCTGGGAAA

28261 GGACCTTGAACAAAGTAAAAGTTTAATGAAGGCGCAGAGGAGTCAGGTTAATTGGCACTG

28321 GAAGATCACCTGCGAGGCAGTAAGATGACGACCCAGTTAGAGAAGCGGTAAAGGACCAAC

28381 AGGCAGGTCACAGCAGAGACTCACGGTCAGTATGAAAAGACTCCTAACCTCACCATGAGT

28441 AACCAAACATCCACACTGAAATCATCAGACGGTGTTTGCCGACAGAGCAGTGTTGCTGTG

28501 GCTGTGGCAATGGGGTACCACATACGGTTGGTTCAGGGTAAGCTGCCTCTGTGAAAAGGC

28561 GATTCGGCAAGATCTATCCACATTAAAACATGCATACCCTTTGATCCAGCAATTCCACTT

28621 GTAGGAATTTATTCTAAAAAGCTCATAAAGATACACGTACAAGGATGTTCACTGCAGCAC

28681 TGCTTGCAATACAAAACCCTGGAAACAACGTGAGCCCATTAATAAGGGGACAATCAAATA

28741 CAGAATGATACTCTTACACACACAAAATAGGGAACAGTCCATAAACACAGGCATCGAGAG

28801 ATGTCCAAGACTATTTTTAAGGAAAAAAAAGTTGCAGAACACTGTAAGAGATTTTGTGCA

28861 AAAGTAAATGAACTACACACTTATGTATGCACACAAATAGCATGTATTTATGTAGGTGAG

28921 GAACGTAGCAGAGGCTGCACCAACCTCCTATTAGGTGCTCCCTCCGGGAGGAGGGCTGGG

28981 AGGATGGCTGGGAAGAGGTGGGCACTTTAAACACTGATTTCCTTTTTAGAATAAGCCTAT

29041 ATTATTGTTCTGACTGAAACCAGACAAAAGTAAAAGTTGGTCTGAAATAACTATCAGATG

29101 CGTTTGCACCTTTAGAACTTCAGGAACGAACGCACGCAGAGGGGATTCCGCCGGGCAGGT

29161 GACTTTTGTTACAGTGTAAGACTTAATGTCTGGGAAAAGCAAA
